# Body Mass Index and All‐Cause, Cancer, and Cardiovascular Mortality in Adults Aged 16–50 Years With and Without Type 2 Diabetes: An Analysis of Primary Care Records in England

**DOI:** 10.1111/dom.70844

**Published:** 2026-05-12

**Authors:** Yijing Chen, Cameron Razieh, Jonathan Goldney, Kamlesh Khunti, Melanie Davies, Francesco Zaccardi, Thomas Yates

**Affiliations:** ^1^ Diabetes Research Centre University of Leicester Leicester UK; ^2^ NIHR Leicester Biomedical Research Centre University Hospitals of Leicester NHS Trust and University of Leicester Leicester UK; ^3^ NIHR Applied Research Collaborations East Midlands University Hospitals of Leicester NHS Trust Leicester UK; ^4^ Leicester Real World Evidence Unit, Diabetes Research Centre University of Leicester Leicester UK

**Keywords:** database research, diabetes complications, observational study, real‐world evidence, type 2 diabetes

## Abstract

**Aims:**

To examine the association between body mass index (BMI) and risk of all‐cause, cancer, and cardiovascular diseases (CVD) mortality among adults aged 16–50 years with and without type 2 diabetes (T2D).

**Materials and Methods:**

We conducted a retrospective matched cohort study using primary care records from the Clinical Practice Research Datalink (CPRD) linked to Hospital Episode Statistics (HES) and Office for National Statistics (ONS) mortality records. Individuals aged 16–50 years with T2D were matched (1: up to 10) to those without T2D by year of birth, sex, and general practice. Using flexible parametric survival models and competing risk approaches, we estimated hazard ratios (HRs) and 10‐year cumulative incidences of all‐cause and cause‐specific mortality adjusted for confounders.

**Results:**

A total of 384 787 individuals were included, of whom 86 606 (22.5%) lived with T2D (median age at diagnosis, 44.0 years). The associations between BMI and all‐cause mortality, as well as cause‐specific mortality, exhibited a U‐shaped pattern, with the nadir occurring at a BMI of 29.3 kg/m^2^ and 27.2 kg/m^2^ in those with and without T2D, respectively. The highest absolute risk and the greatest risk differences between individuals with and without T2D were observed at lower BMI values. At 45 years and BMI of 20 kg/m^2^, the 10‐year cumulative incidence of all‐cause mortality risk was 17.3% (95% CI: 13.9%, 20.6%) for individuals with T2D compared to 4.4% (95% CI: 4.0%, 4.7%) in those without.

**Conclusions:**

The excess risk of mortality associated with T2D was greatest at lower BMI, where absolute risk estimates were high.

## 1 Introduction

Diabetes now affects at least 5% of the global population, with approximately 90% of those individuals living with type 2 diabetes (T2D) [1, 2]. Although T2D has historically been associated with older age, recent years have seen a rapid rise in prevalence at younger ages [3, 4, 5]. The onset of T2D in younger adults is characterised by more severe disease progression, faster development of insulin resistance, and a higher relative risk of cardiovascular disease (CVD), with severe obesity likely contributing to these outcomes [5, 6, 7, 8]. Body mass index (BMI), a common marker of adiposity used to classify obesity in routine clinical practice, is linked to mortality events, including all‐cause, cancer, and cardiovascular disease‐related deaths [9, 10, 11]. Excess adiposity has been associated with multiple metabolic, inflammatory, and hormonal pathways that contribute to CVD and certain cancers [12]. Previous studies suggest that the association between BMI and mortality is strongest at younger ages, highlighting the importance of BMI as a risk factor for mortality among this high‐risk group [13, 14, 15]. Furthermore, the impact of T2D on mortality risk may also vary across age groups, with relatively greater excess risks reported at younger ages compared with older populations [16]. Research in older adults with T2D typically reveals a J‐shaped association between BMI and mortality [17, 18, 19, 20], however, there is a lack of evidence for the association between BMI and mortality in young‐to‐middle‐aged adults with T2D.

This study aims to investigate the association between BMI and all‐cause, cancer, and CVD mortality in adults aged 16–50 years with and without T2D to help inform clinical management for this high‐risk population.

## 2 Materials and Methods

### 2.1 Data Source

This study used data extracted from Clinical Practice Research Datalink (CPRD) GOLD and Aurum databases, linked to Hospital Episode Statistics (HES) dataset and Office for National Statistics (ONS) for demographic, clinical, and mortality data. This study has been approved by CPRD Independent Scientific Advisory Committee (ISAC; protocol number 20_000288) [21] and has been conducted in line with the RECORD guidelines for conducting and reporting observational studies using routinely collected electronic health records (checklist in the Table S10).

### 2.2 Study Population

We conducted a retrospective matched cohort study that included all individuals aged 16–50 years with a first diagnosis of T2D between 1 January 2000 and 31 October 2020 in CPRD GOLD or Aurum, with linkage to HES and ONS mortality data. Individuals with T2D were identified using clinical codes consistent with the prior CPRD study [21]. Up to 10 patients living without a T2D diagnosis before the study end date were matched to the individuals with T2D by year of birth, sex, and general practice without replacement. For individuals with T2D, the index date was their diagnosis date; matched individuals without T2D were assigned the same index date. The full data extraction process has been described previously [21]. Briefly, individuals were excluded if they had duplicate records across CPRD GOLD and Aurum, lacked linkage to HES or ONS, had a prior diagnosis of cancer or cardiorenal disease (defined as ischaemic heart disease, stroke, chronic kidney disease, or receipt of renal replacement therapy) at or before the index date, or had missing data on sex, ethnicity, deprivation status, or smoking. For this study, individuals with missing BMI values were additionally excluded. BMI was missing in 19.0% of those with T2D and 63.4% of those without. The final study population was restricted to individuals with a BMI ranging from 18.5 to 60 based on the 1st to the 99th percentile of BMI to exclude extreme values that could reflect coding errors. Figure S1 displays the cohort selection process.

### 2.3 Exposure, Outcome and Confounders

The exposure was the most recent BMI recorded within 5 years before the index date. BMI was defined as weight in kilograms divided by height in metres squared (kg/m^2^). The primary outcomes were all‐cause and cause‐specific mortality, obtained from ONS. Cause‐specific mortality was categorised into cancer (ICD‐10: C00–C97), cardiovascular (I00–I99), and other causes (non‐cancer/non‐cardiovascular) mortality.

Age at index date, sex, smoking status, hypertension (recorded at or up to 5 years before the index date), and prescriptions for lipid‐lowering and anti‐hypertensive medications (in the year before the index date) were extracted from CPRD. Ethnicity (White, Black [Black African, Black Caribbean, Black Other], South Asian [Bangladeshi, Indian, Pakistani] and Other [Chinese, Other Asian, Mixed, Other]) was derived from HES; and deprivation status (ranging from quintile 1—least deprived to quintile 5—most deprived) was extracted from the Index of Multiple Deprivation (IMD) 2010 data. Alcohol‐related disorders were identified using Read/SNOMED code lists from validated sources, including the ClinicalCodes.org repository, opencodelists.org repository, prior literature, and GitHub (Table S7).

### 2.4 Statistical Analysis

For the descriptive analyses, BMI was categorised into three groups using standard ethnic cut‐points: healthy (White ≥ 18.5 to < 25 kg/m^2^; Non‐White ≥ 18.5 to < 23 kg/m^2^), overweight (White ≥ 25 to < 30 kg/m^2^; Non‐White ≥ 23 to < 27.5 kg/m^2^) and obese (White ≥ 30 kg/m^2^; Non‐White ≥ 27.5 kg/m^2^), based on WHO recommendations and UK NICE guidance [22, 23]. All‐cause and cause‐specific mortality rates across different BMI groups among people with and without T2D were estimated by the number of deaths and the total person‐years at risk. In addition, among individuals with T2D, the distributions of BMI at index date between those with and without alcohol‐related disorders were compared. Complete‐case Royston‐Parmar flexible parametric models, with follow‐up years as the underlying time scale, were used to estimate the relative hazard (e.g., hazard ratios [HRs]) and absolute risks (e.g., cumulative incidence functions [CIFs]) of all‐cause and cause‐specific mortality. An interaction term between three variables (BMI, age at index date and T2D status) was included in the model. Both BMI and age at index date were modelled with natural cubic spline functions to capture their respective non‐linear relationships with mortality [21, 24]. Different degrees of freedom for the baseline hazard function and spline functions of BMI and age at index were initially compared, with the final models selected using the Bayesian Information Criterion (BIC). Using this approach, two internal knots (placed at the 33rd and 66th percentiles, along with boundary knots at the maximum and minimum values) were used for the baseline hazard function, as well as for the spline functions of BMI and age at index. Sex, ethnicity, deprivation, smoking status, hypertension, and use of medications were included as potential confounders. Hazard ratios (i.e., HRs) of all‐cause and cause‐specific mortality (cancer, CVD, and other causes) were estimated across BMI value for individuals with and without T2D, using individuals without T2D with a BMI of 25 kg/m^2^ as the reference group (i.e., HR = 1). Ten‐year absolute risks (i.e., cumulative incidences) for all‐cause mortality were also estimated for individuals with and without T2D across different BMI values. Estimates for both HRs and 10‐year absolute risks were calculated at index age of 40, 45 (approximately the median of the age distribution) and 50 years.

We analysed competing risks using a cause‐specific hazard framework [25]. Separate flexible survival models were fitted for the cause‐specific hazards of the event of interest and each competing event, treating competing events as censoring within each model [26]. Cumulative incidence functions were then derived from these hazards to estimate absolute risks at 10 years across BMI values, stratified by T2D status and age at the index date, by combining the cause‐specific hazard of the event of interest with those of the competing events over time [27]. Restricted mean survival time (RMST) over a 10‐year horizon was additionally estimated from the fitted flexible parametric survival models across BMI values at index ages of 40, 45, and 50 years.

To examine the robustness of the findings, multiple sensitivity analyses were conducted. HRs among the men and women subgroups were examined to explore whether findings differed by sex. The analysis was repeated after restricting to non‐smokers to explore the potential for collider bias, as previously suggested [19, 28, 29]. People who died within 2 follow‐up years and 5 follow‐up years were removed from the analysis to investigate reverse causality. The potential for overadjustment with mediating variables was explored by re‐estimating the models without including hypertension, antihypertensive treatment, and lipid‐lowering medication use. Analyses were repeated after excluding individuals with records of alcohol‐related conditions to account for potential confounding by alcohol use. Lastly, analyses were repeated following multiple imputation of BMI (10 imputed datasets using Rubin's rule) in individuals with T2D. Imputation was restricted to the T2D cohort due to the high missingness of BMI in individuals without T2D.

All analyses were conducted using Stata version 18 and R version 4.3.1 on the ALICE High Performance Computing Platform at the University of Leicester.

## 3 Results

### 3.1 Baseline Characteristics

A total of 384 787 individuals were included in the study, of which 86 606 were newly diagnosed with T2D. The median (IQR) BMI was 33.9 kg/m^2^ (29.4, 39.3) in individuals with T2D compared with 26.6 kg/m^2^ (23.5, 30.4) in those without, with 77.90% of the T2D cohort living with obesity, compared with 23.90% in those without T2D. Individuals with T2D were also more likely to be men (52.6% vs. 47.8%) and from the most deprived quintile (39.5% vs. 34.1%), whilst less likely to be of White ethnicity (67.1% vs. 78.8%). The distribution of demographic and clinical characteristics across categories of BMI and T2D status is reported in Table 1. Alcohol‐related disorders were more common among individuals with T2D, particularly at lower BMI (accounting for 18.3% in those with T2D and normal weight). Figure S2 shows the BMI distribution in those with and without alcohol related disorders. Comparisons between individuals included in the analytic cohort and those excluded due to missing BMI are presented in Table S8. Individuals with recorded BMI had a higher prevalence of cardiometabolic risk factors compared with those without BMI records, particularly among those without T2D.

**TABLE 1:** Baseline characteristics and mortality outcomes stratified jointly by BMI category (normal weight, overweight, obesity) and T2D status.

	No T2D			T2D		
	Normal (*N* = 99 223)	Overweight (*N* = 106 977)	Obesity (*N* = 91 981)	Normal (*N* = 3658)	Overweight (*N* = 15 479)	Obesity (*N* = 67 469)
Sex (%)						
Men	39 082 (39.4)	60 194 (56.3)	43 277 (47.1)	2192 (59.9)	10 017 (64.7)	33 311 (49.4)
Women	60 141 (60.6)	46 783 (43.7)	48 704 (53.0)	1466 (40.1)	5462 (35.3)	34 158 (50.6)
Age at index date						
Mean (SD)	41.1 (7.31)	42.9 (6.25)	43.3 (6.06)	42.0 (6.88)	43.1 (6.03)	42.3 (6.49)
Median [Min, Max]	43.0 [16.0, 50.0]	45.0 [16.0, 50.0]	45.0 [16.0, 50.0]	44.0 [16.0, 50.0]	45.0 [16.0, 50.0]	44.0 [16.0, 50.0]
Deprivation Index (%)						
1 (least deprived)	16 372 (16.5)	15 847 (14.8)	10 316 (11.2)	454 (12.4)	1843 (11.9)	7145 (10.6)
2	16 828 (17.0)	17 204 (16.1)	12 296 (13.4)	470 (12.8)	2035 (13.1)	8874 (13.2)
3	17 958 (18.1)	19 521 (18.2)	15 728 (17.1)	641 (17.5)	2619 (16.9)	11 477 (17.0)
4	21 789 (22.0)	24 282 (22.7)	22 197 (24.1)	879 (24.0)	3824 (24.7)	16 807 (24.9)
5 (most deprived)	26 276 (26.5)	30 123 (28.2)	31 444 (34.2)	1214 (33.2)	5158 (33.3)	23 166 (34.3)
Ethnicity (%)						
White	86 683 (87.4)	82 765 (77.4)	65 606 (71.3)	2408 (65.8)	8921 (57.6)	46 782 (69.3)
Black	2675 (2.7)	6145 (5.7)	9314 (10.1)	207 (5.7)	1151 (7.4)	5903 (8.7)
Other	4791 (4.8)	7933 (7.4)	7301 (7.9)	364 (10.0)	1865 (12.0)	5454 (8.1)
South Asian	5074 (5.1)	10 134 (9.5)	9760 (10.6)	679 (18.6)	3542 (22.9)	9330 (13.8)
Smoke–yes (%)	28 078 (28.3)	25 394 (23.5)	17 718 (21.7)	1161 (31.7)	4081 (26.4)	17 234 (25.5)
Lipid lowering–yes (%)	1581 (1.6)	4815 (4.5)	6903 (7.5)	585 (16.0)	3494 (22.6)	13 669 (20.3)
Anti‐hypertension–yes (%)	5439 (5.5)	10 456 (9.8)	15 054 (18.5)	1110 (19.2)	4747 (29.5)	21 594 (34.6)
Hypertension–yes (%)	625 (0.6)	1250 (1.2)	1522 (1.9)	62 (1.7)	411 (2.7)	1902 (2.8)
Alcohol‐related disorder (%)	3 (0.0)	0 (0.0)	1 (0.0)	739 (18.3)	1104 (6.9)	2769 (4.1)
Total person years	1037648.80	1083889.45	865540.80	35237.36	150225.46	599388.24
N. deaths						
All‐Cause	3494	3042	2984	383	807	3147
Cancer	1274	1198	1035	70	256	864
Cardiovascular	495	638	807	66	190	997
Other	1725	1206	1142	247	361	1286
Mortality rate (95% CI) [Per 1000 person‐years]						
All‐Cause	3.37 (3.26, 3.48)	2.81 (2.71, 2.91)	3.45 (3.32, 3.57)	10.87 (9.79, 11.98)	5.37 (5.01, 5.74)	5.25 (5.07, 5.43)
Cancer	1.23 (1.16, 1.30)	1.11 (1.04, 1.17)	1.20 (1.12, 1.27)	1.99 (1.53, 2.47)	1.70 (1.50, 1.92)	1.44 (1.35, 1.54)
Cardiovascular	0.48 (0.44, 0.52)	0.59 (0.54, 0.63)	0.93 (0.87, 1.00)	1.87 (1.45, 2.33)	1.26 (1.09, 1.44)	1.66 (1.56, 1.77)
Other	1.66 (1.58, 1.74)	1.11 (1.05, 1.18)	1.32 (1.24, 1.40)	7.01 (6.16, 7.89)	2.40 (2.16, 2.66)	2.15 (2.03, 2.26)

Abbreviations: T2D, type 2 diabetes; CI, confidence interval; SD, standard deviation.

### 3.2 Mortality Rates

The median follow‐up time was 8.7 years for individuals with T2D and 10.0 years for those without, with a total of 3 771 930 person‐years of observations across the cohort. During follow‐up, 4337 (5.0%) deaths occurred in those with T2D, of which 1190 (27.4% of all deaths), 1253 (28.9%), and 1894 (43.7%) were cancer‐related, CVD‐related, and due to other causes, respectively (Table 1); corresponding figures in individuals without T2D were 9520 (3.2%) deaths, of which 3507 (36.8% of all deaths), 1940 (20.4%) and 4073 (42.8%) were cancer‐related, CVD‐related, and due to other causes, respectively.

In individuals with T2D, the mortality rates for all causes, cancer, CVD, and other causes were 5.53 (95% CI: 5.36, 5.69), 1.52 (1.43, 1.60), 1.60 (1.51, 1.69), and 2.41 (2.30, 2.52) per 1000 person‐years, respectively. In those without T2D, corresponding rates were 3.19 (95% CI: 3.12, 3.25), 1.17 (1.14, 1.21), 0.65 (0.62, 0.68), and 1.36 (1.32, 1.41), respectively.

Alcoholic liver disease and pulmonary diseases were the most common causes of death among individuals classified as dying from other causes, each in the overall cohorts of those with and without T2D (Table S1) and in the subset with BMI < 25 kg/m^2^ (Table S2).

### 3.3 Relative Risks

**FIGURE 1:**
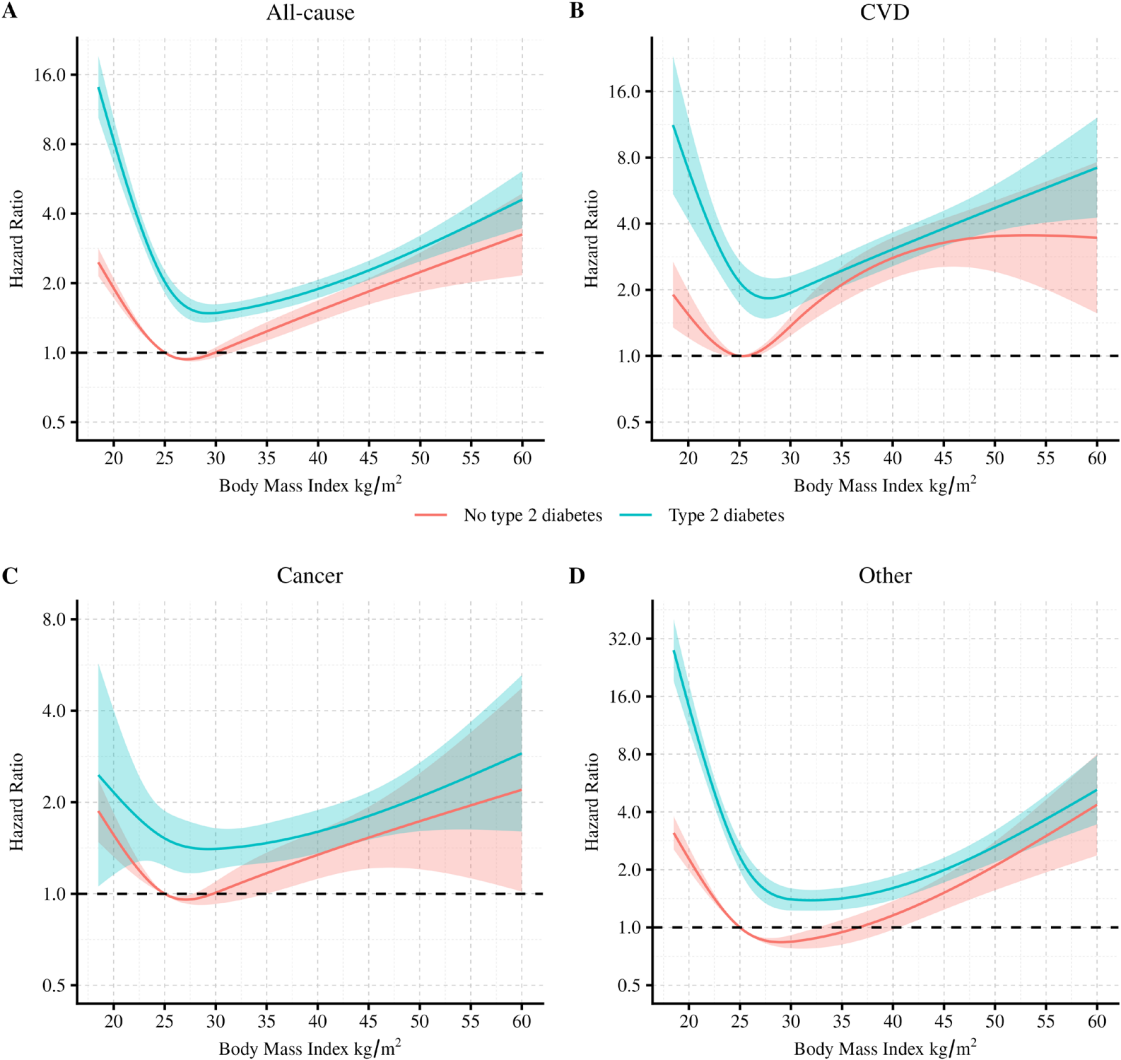
Hazard Ratios (HRs) for mortality by BMI and T2D status. The panels show the HRs for all‐cause mortality (Panel A), CVD mortality (Panel B), cancer mortality (Panel C), mortality due to other causes (Panel D) in individuals with and without type 2 diabetes (T2D), at index ages of 45 years, across different BMI values compared to the reference group without T2D at a BMI of 25 kg/m^2^. Shaded areas show 95% confidence intervals. HRs were adjusted for sex, ethnicity, deprivation and smoking status, use of lipid‐lowering and anti‐hypertension medications, hypertension status, and were stratified by T2D status and modelled at index age of 45 years.

A joint Wald test showed there was statistically significant evidence to reject the null hypothesis of no three‐way interaction between BMI, age, and T2D status ($\chi^{2}\left(9\right)=21.75,p=0.0097$), indicating the presence of a significant effect modification. The adjusted HRs for all‐cause mortality, comparing individuals at different BMI values to the reference group of individuals without T2D and BMI of 25 kg/m^2^, demonstrated a U‐shaped relationship (Figure 1 and Figure S3). Among individuals aged 45 years at the index date, the nadir of HRs occurred at a BMI of 29.3 kg/m^2^ and 27.2 kg/m^2^ for those with and without T2D, respectively (Figure 1), with consistent findings across other ages (40 and 50 years) (Figure S3). Differences in the magnitude of HRs for all‐cause mortality between individuals with and without T2D was greater at lower BMI values; at a BMI of 20 kg/m^2^ and age of 45 years, the HR was 8.29 (95% CI: 6.61, 10.40) in individuals with T2D vs. 1.90 (1.72, 2.09) in those without (Figure 1; Table S3), compared to HR 1.49 (1.36, 1.62) with T2D versus 0.97 (0.91, 1.03) without T2D at a BMI of 30 kg/m^2^. The attenuation in HR differences between individuals with and without T2D became more apparent at higher BMI levels; for individuals aged 45 years with a BMI of 40 kg/m^2^, the HR was 1.89 (1.72, 2.07) in those with T2D and 1.51 (1.36, 1.69) in those without (Figure 1). Results for age at index of 40 and 50 years were largely consistent to those at age 45 years, although differences between those with and without T2D at lower BMI were smaller at age 50 years (Figure S3 and Table S3).

The pattern observed for all‐cause mortality was mirrored by CVD, cancer, and other causes of death. However, the HRs comparing those with and without T2D for a given value of BMI varied by cause of death, being lowest for cancer mortality and greatest for other causes of death across all ages (Figure 1 and Figure S3).

### 3.4 Absolute Risks

**FIGURE 2:**
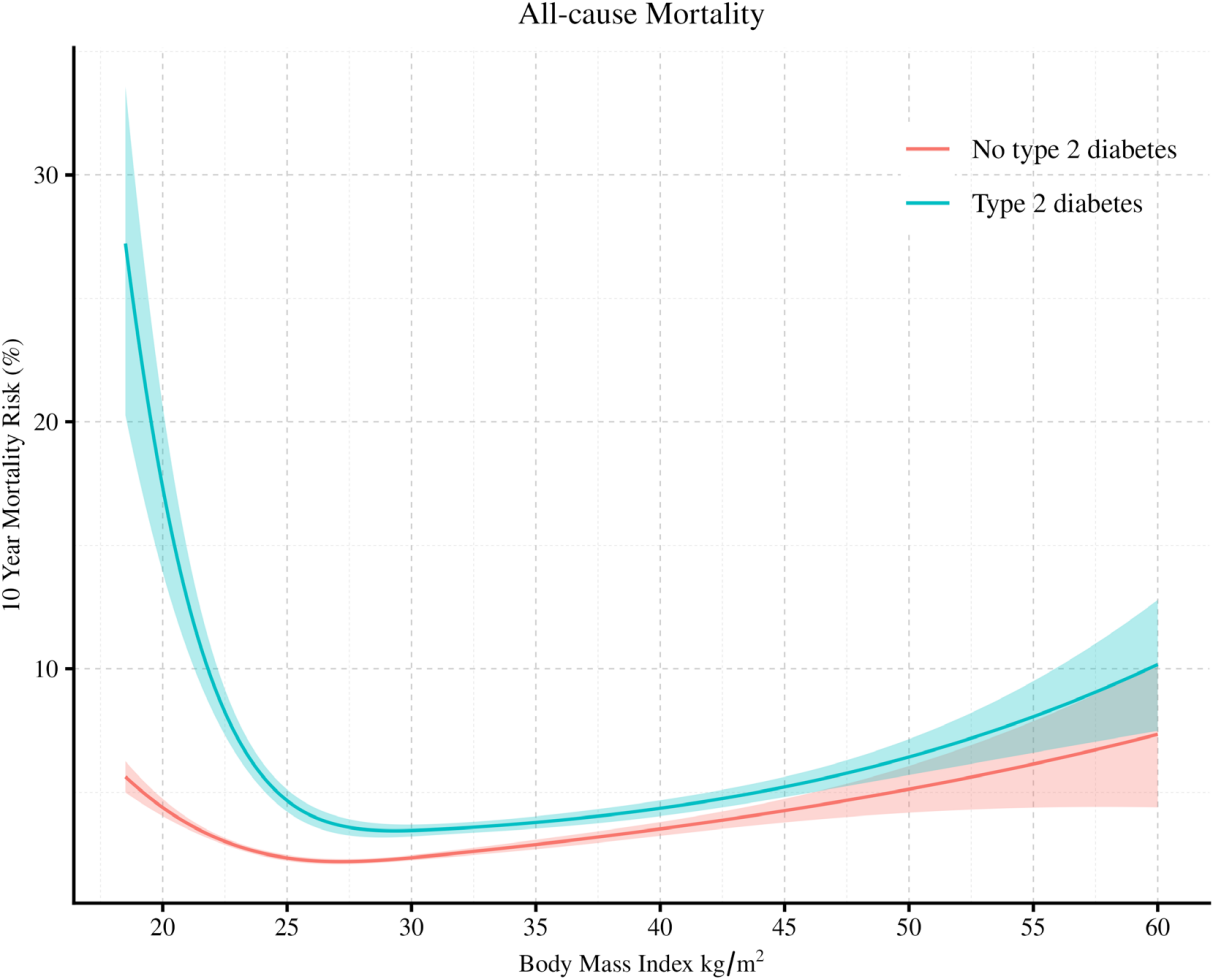
Ten‐year risk (cumulative incidence, %) of all‐cause mortality across BMI and T2D status. The panel shows the 10‐year cumulative incidences of all‐cause mortality in individuals with and without T2D, at index ages of 45 years, across different BMI values. Shaded areas show 95% confidence intervals. Ten‐year cumulative incidences were adjusted for sex, ethnicity, deprivation status, smoking status, use of lipid‐lowering and anti‐hypertension medications, hypertension status, and were stratified by T2D status and modelled at index age of 45 years.

**FIGURE 3:**
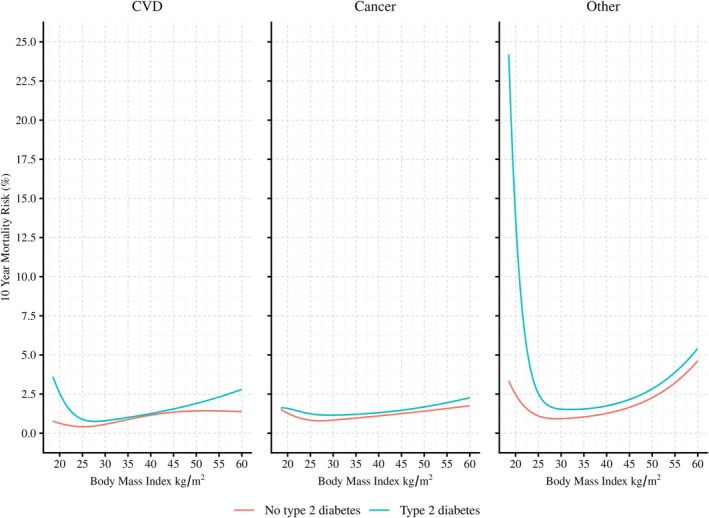
Ten‐year competing risk of cause‐specific mortality across BMI, stratified by BMI and T2D status. The panels show the 10‐year cumulative incidences (%) of CVD mortality, cancer mortality, mortality due to other causes in individuals with and without T2D, at index ages of 45 years, across different BMI values. Ten‐year cumulative incidences were adjusted for sex, ethnicity, deprivation and smoking status, use of lipid‐lowering and anti‐hypertension medications, hypertension status, and were stratified by T2D status and modelled at index age of 45 years.

Absolute risks of all‐cause mortality are shown in Figure 2 (for age 45 years) and Figure S4 (for ages 40 and 50 years); high risk was observed in those with T2D at low BMI. At a BMI of 20 kg/m^2^, the 10‐year mortality risk was 17.3% (95% CI: 13.9%, 20.6%) for individuals diagnosed with T2D at 45 years old, compared to 4.4% (95% CI: 4.0%, 4.7%) in those without T2D (Figure 2, Table S4). At a BMI of 30 kg/m^2^, the 10‐year mortality risk was 3.4% (95% CI: 3.2%, 3.7%) for individuals diagnosed with T2D at 45 years, compared to 2.4% (95% CI: 2.2%, 2.5%) for individuals without T2D. At BMI 40 kg/m^2^, the corresponding figures were 4.4% (95% CI: 4.0%, 4.7%) and 3.5% (95% CI: 3.2%, 3.8%), respectively. For individuals at 40 years, the 10‐year mortality risks tended to be lower across all BMI levels, with larger absolute differences comparing those with versus without T2D at lower BMI values (Figure S4 and Table S4). Conversely, those diagnosed with T2D at 50 years showed higher absolute mortality risks but smaller differences in those with versus without T2D (Figure S4 and Table S4). RMST analyses showed shorter expected survival time among individuals with T2D compared with those without T2D across BMI levels, with largest differences observed at lower BMI values. Over 10 years, at a BMI of 20 kg/m^2^, the RMST was 0.57 years (95% CI: 0.42, 0.73), compared to −0.04 years (−0.07, −0.01) at a BMI of 45 kg/m^2^ Table S6. The 10‐year mortality risk for cause‐specific mortality showed a similar U‐shaped pattern to all‐cause mortality (Figure 3; Figure S5). At a BMI of 20 kg/m^2^, the 10‐year absolute risk of dying from CVD, cancer, and other causes were 2.6%, 1.6%, and 13.8% in individuals diagnosed with T2D at 45 years old, compared to 0.6%, 1.3%, and 2.5% in non‐T2D individuals (Table S5). At BMI 30 kg/m^2^, the corresponding risks were lower at 0.8%, 1.2%, and 1.5% for individuals with T2D, compared to 0.6%, 0.8%, and 0.9% in those without T2D. Beyond BMI 30 kg/m^2^, mortality risks for cause‐specific deaths generally increased, but with the differences between those with and without T2D decreasing. At BMI 40 kg/m^2^, the absolute risks for cancer, CVD, and other‐causes mortality were 1.3%, 1.3%, and 1.8% for individuals diagnosed with T2D at 45 years old, compared to 1.2%, 1.1%, and 1.3% for those without T2D. The overall pattern and equivalent values for those at age 40 and 50 years are displayed in Figure S5, and Table S5.

### 3.5 Sensitivity Analysis

Stratifying by sex, the HRs for all‐cause and cause‐specific mortality in individuals with and without T2D showed largely consistent patterns with the primary analysis, except for women diagnosed with T2D at age 45 showing a weaker U‐shape relationship between BMI and cancer mortality (Figure S6); however, the HRs comparing those with and without T2D at low BMI tended to be greater in women than men. After removing the mortality cases within the first 2 and 5 years of follow‐up, the pattern of results of HRs was similar to that of the main analysis (Figures S7 and S8). The pattern of associations between BMI and mortality risk persisted when potential mediators, including hypertension and related medications, were excluded from the model (Figure S9). The U‐shaped associations remained largely unchanged after excluding individuals with alcohol‐related conditions (Figure S10). Although excluding smokers led to some attenuation in the associations with CVD mortality, the overall interpretation remained unchanged, and results for other outcomes were robust (Figure S11). Compared to the complete case analysis, the results of HRs were unchanged when imputing missing BMI values in those with T2D (Figure S12).

## 4 Discussion

### 4.1 Main Findings

Adults with T2D diagnosed at younger ages exhibited disproportionately higher mortality risk at lower BMI (< 25 kg/m^2^), with the 10‐year risk of all‐cause mortality reaching 17.3% at a BMI of 20 kg/m^2^ in those aged 45 years at diagnosis, largely driven by non‐CVD and non‐cancer mortality. Conversely, both individuals with and without T2D experienced elevated and gradually converging mortality risks at high BMI. To our knowledge, this is the first large‐scale study to comprehensively examine BMI‐associated mortality patterns in young‐to‐middle aged adults with and without T2D, identifying a particularly vulnerable group: individuals with low BMI and early‐onset T2D.

### 4.2 Comparison to Existing Literature

Our findings are broadly consistent with a previous UK study using CPRD data that similarly demonstrated an “obesity paradox” in a general cohort of newly diagnosed T2D, where those with higher BMI had lower mortality risk compared to those with normal weight [30]. Previous research in individuals with and without T2D from the United States and Japan has also reported that those with T2D at normal or lower BMI values face higher mortality rates or higher all‐cause mortality risk, compared with those without T2D [31], including those under 65 years of age [20]. Our study adds to this existing evidence by showing that the higher risk at lower BMI may be particularly pronounced at younger ages. The mortality risk difference at lower BMI values between individuals with and without T2D was greater in those diagnosed with T2D at a relatively younger age (e.g., 40–45 years), as compared to those diagnosed later (50 years), indicating that the impact of T2D on mortality may be particularly relevant to individuals diagnosed at younger ages with normal or lower BMI. This finding was consistent with a study in Sweden, which reported that HRs increased at lower BMI values among younger individuals with T2D [32]. Although most previous studies have focused on hazard ratios (HRs) to assess the mortality risk associated with BMI in individuals with and without T2D, our study further provides evidence by estimating the 10‐year absolute mortality risks. We observed that absolute differences between individuals with and without T2D were largest at lower BMI values, especially among young‐to‐middle‐aged adults, with risks exceeding 17% at low BMI.

### 4.3 Potential Mechanisms

The high mortality risk at low BMI in those with T2D could be attributed to biological/mechanistic processes and/or data/analysis methods. Biologically, there are differences in the mechanisms and subtypes of T2D between individuals with obesity and those with normal weight [17, 33, 34], which may lead to more severe complications and higher mortality risks for individuals developing T2D despite having a normal BMI [35]. For example, Ahlqvist et al. defined subtypes of diabetes [36]: although mild obesity related diabetes (MOD) and severe insulin‐deficient diabetes (SIDD) are both associated with younger age, SIDD is emerging as having a higher risk of adverse outcomes despite being associated with lower BMI [37, 38]. However, given the markedly elevated mortality risk observed at lower BMI values in individuals with T2D was largely driven by non‐cardiovascular and non‐cancer deaths, biological/mechanistic processes and/or data/analysis methods beyond classical atherosclerotic may be required to explain the excess risk in lean individuals with T2D, which warrants further investigation. Alternatively, one of the most common explanations for the U‐shaped association between BMI and health outcomes is reverse causation, where underlying disease processes or unhealthy behaviours lead to weight loss, which is associated with a higher mortality risk [39]. However, the persistence of associations after excluding early deaths suggests that reverse causation alone is unlikely to fully explain the findings. Recently, the International Diabetes Federation has introduced the concept of type 5 diabetes, a form of diabetes associated with chronic undernutrition, which may be misclassified as T2D and thus contribute to excess mortality in lean individuals [40]. Alcohol‐related and substance‐use disorders may also contribute to the excess mortality observed in younger adults with T2D and low BMI [41]. In our cohort, alcohol‐related disorders were more prevalent among individuals with T2D at lower BMI, and alcoholic liver disease and pulmonary diseases were prominent causes of non‐CVD/non‐cancer mortality. Although exclusion of individuals with alcohol‐related conditions did not significantly alter the results, these findings suggest that comorbid substance‐related disease may partially contribute to the elevated risk in this subgroup. Collider bias due to confounders such as smoking or pre‐existing CVD has also been considered as a potential explanation [42]. However, previous investigations have suggested that this is unlikely to fully account for the observed U‐shaped association [43], with a further study showing the high risk at low BMI in a general cohort of T2DM with and without CVD persists across a range of analytical approaches to account for collider bias [31], a conclusion further supported by the exclusion criteria (e.g., prevalent CVD) and sensitivity analyses conducted for the present analysis. Another possible explanation for the higher mortality risk among individuals with T2D at lower BMI values is selection bias due to missingness of BMI data. In our study population, individuals with recorded BMI had a higher prevalence of recorded smoking, antihypertensive medicine, lipid‐lowering medicine, and hypertension. In primary care, BMI is more likely to be measured during consultations for other risk factors. This pattern was most evident in those without T2D, who accounted for the majority of missing BMI values. Under these circumstances, restricting analyses to individuals with recorded BMI could preferentially retain non‐T2D individuals with a higher prevalence of clinical risk factors, which might inflate mortality estimates in the analysed non‐T2D group and attenuate comparisons with T2D. However, the direction and magnitude of this bias cannot be determined with certainty, as BMI recording may also be influenced by other clinical processes that are not fully captured in the available data. However, this is unlikely to fully account for the high risk at low BMI observed in those with T2D, where missingness was lower and where associations persisted after multiple imputation. Lastly, misclassification of diabetes type may have contributed to the high risk at low BMI [44].

At higher BMI values, mortality risks gradually converged between individuals with and without T2D, which contrasts with a large population‐based Swedish cohort (average age, 60 years) that found excess CVD mortality associated with T2D at BMI values > 40 kg/m^2^ [32]. It is possible that extreme obesity at a young age is more strongly associated with cardiometabolic dysfunction than at older age, potentially attenuating the specific impact of diabetes on CVDs and related complications [45, 46].

### 4.4 Strength and Limitations

The study had several strengths, including its use of primary care data, a large sample size, and an extended follow‐up period. Its focus on young‐to‐middle age adults with T2D addresses an under‐researched population with significant clinical needs [47]. However, several limitations remained. The high proportion of missing BMI data among individuals without T2D may have introduced selection bias. As discussed, missingness was substantially lower in the T2D population, but among those without T2D, BMI recording appeared to be associated with a higher prevalence of recorded cardiometabolic risk factors, suggesting that the non‐T2D group included in the analysis represents a higher‐risk subset. Then, leaner and potentially healthier individuals without T2D may be underrepresented, so comparisons between those with and without T2D should be interpreted cautiously. As discussed, the pattern of association between BMI and mortality, particularly in those with T2D, may be due to reverse causation or collider bias. Although our sensitivity analysis suggested a possible role of smoking in biasing associations with CVD mortality, other outcomes and sensitivity analysis remained robust. Although we used validated clinical code lists consistent with previous studies on the same CPRD dataset [21], some misclassification between type 1 diabetes and T2D may have occurred, particularly among younger lean adults. However, such misclassification is unlikely to fully explain the observed BMI–mortality patterns. BMI was assessed at a single time point prior to the index date and may not fully capture long‐term exposure to excess body weight. In addition, BMI does not distinguish between fat and lean mass or capture body fat distribution, which may not fully reflect underlying body composition and metabolic risk. Nonetheless, given the observational nature of the study, causal associations cannot be established. Information on certain lifestyle factors, such as diet and physical activity, was not available in the dataset and therefore, residual confounding cannot be fully excluded. Furthermore, due to the required computational resources, confidence intervals for the 10‐year cause‐specific mortality risks were not estimated.

### 4.5 Conclusions

This study identified a U‐shaped relationship between BMI and risk of all‐cause and cause‐specific mortality among adults aged 16–50 years, with the highest risk observed in individuals with T2D at lower BMI, where absolute mortality risks reached levels that are unexpectedly high for adults in this age range, exceeding 17% over 10 years. Mortality differences between those with and without T2D narrowed with increasing BMI. Our findings suggest that the excess risk of death for individuals diagnosed with T2D at younger ages and low BMI is substantial and may translate into significantly reduced life expectancy. This may highlight a particularly high‐risk group that warrants further research into underlying mechanisms and targeted public health strategies.

## Author Contributions

T.Y., F.Z. and C.R. were involved in the conception and development of the research question. F.Z. acquired the data with support from C.R. Y.C. led the analysis, with support from F.Z., T.Y. and C.R. Y.C. drafted the manuscript. All authors contributed to the interpretation and edited and revised the manuscript for important intellectual content. All authors approved the final version of the manuscript. T.Y. and F.Z. are the guarantors of this work and, as such, had full access to all the data in the study and take responsibility for the integrity of the data and the accuracy of the data analysis.

## Funding

This work was supported by Wellcome Trust, NIHR Leicester BRC, NIHR ARC‐EM, and Leicester BHF CRE.

## Conflicts of Interest

F.Z. Consultancy for Servier, Menarini, Daiichi‐Sankyo. All authors declare no conflicts of interest. T.Y. Consultancy for Regeneron, research funding from Biomea Fusion.

## Supporting information


**Figure S1:** Study Population Selection Process Flow Chart. The overall selection process has been described previously (1). In the present study, only the additional selection criteria specific to this analysis are illustrated.
**Figure S2:** Distribution of body mass index (BMI) in individuals with type 2 diabetes, comparing those with and without alcohol‐related disorders.
**Figure S3:** Hazard Ratios (HRs) for mortality by BMI, T2D status, and age at index date. The panels show the HRs for all‐cause mortality (Panel A), CVD mortality (Panel B), cancer mortality (Panel C), mortality due to other causes (Panel D) in individuals with and without type 2 diabetes (T2D), at index ages of 40 and 50 years, across different BMI values compared to the reference group without T2D at a BMI of 25 kg/m^2^. Shaded areas show 95% confidence intervals. HRs were adjusted for sex, ethnicity, deprivation and smoking status, use of lipid‐lowering and anti‐hypertension medications, hypertension status, and were stratified by T2D status and modelled at index ages of 40 and 50 years.
**Figure S4:** 10 year risks (cumulative incidence, %) of all‐cause mortality across BMI, stratified by age (40 and 50 years at index date) and T2D status. Shaded areas show 95% confidence intervals. 10‐year cumulative incidences were adjusted for sex, ethnicity, deprivation and smoking status, use of lipid‐lowering and anti‐hypertension medications, hypertension status, and were stratifiehd by T2D status and modelled at index ages of 40 and 50 years.
**Figure S5:** 10‐year competing risk of cause‐specific mortality across BMI, stratified by T2D status. The panels show the 10‐year risks (cumulative incidences, %) of CVD mortality, cancer mortality, mortality due to other causes in individuals with and without T2D, modelled at index ages of 40 (Panel A) and 50 years (Panel B), across different BMI values. 10‐year cumulative incidences were adjusted for sex, ethnicity, deprivation and smoking status, use of lipid‐lowering and anti‐hypertension medications, and hypertension status, and were stratified by T2D status and modelled at index ages of 40 and 50 years.
**Figure S6:** Hazard Ratios (HRs) for mortality by BMI, T2D status, and age at index date among male and female subgroups. The panels show the HRs for all‐cause mortality (Panel A), CVD mortality (Panel B), cancer mortality (Panel C), and mortality due to other causes (Panel D) with and without T2D across different BMI values compared to the reference group without T2D at a BMI of 25 kg/m2. Results are presented separately for males and females and further modelled at age at index date (40, 45, and 50 years). Shaded areas show 95% confidence intervals. HRs were adjusted for sex, ethnicity, deprivation, smoking status, use of lipid‐lowering and anti‐hypertension medications, and hypertension status, and were stratified by T2D status and modelled at index ages of 40, 45, and 50 years.
**Figure S7:** Hazard ratios (HRs) for Mortality by BMI, T2D status, and age at index date after excluding mortality cases less than 2‐year follow‐up. The panels show the HRs for all‐cause mortality (Panel A), CVD mortality (Panel B), cancer mortality (Panel C), and mortality due to other causes (Panel D) in individuals alive more than 2 follow‐up years with and without T2D across different BMI values compared to the reference group without T2D at a BMI of 25 kg/m^2^. Results are modelled at age at index date (40, 45, and 50 years). Shaded areas show 95% confidence intervals. HRs were adjusted for sex, ethnicity, deprivation, smoking status, use of lipid‐lowering and anti‐hypertension medications, and hypertension status, and were stratified by T2D status and modelled at index ages of 40, 45, and 50 years.
**Figure S8:** Hazard ratios (HRs) for Mortality by BMI, T2D status, and age at index date after excluding mortality cases less than 5‐year follow‐up. The panels show the HRs for all‐cause mortality (Panel A), CVD mortality (Panel B), cancer mortality (Panel C), and mortality due to other causes (Panel D) in individuals alive more than 5 follow‐up years with and without T2D across different BMI values compared to the reference group without T2D at a BMI of 25 kg/m^2^. Results are modelled at age at index date (40, 45, and 50 years). Shaded areas show 95% confidence intervals. HRs were adjusted for sex, ethnicity, deprivation, smoking status, use of lipid‐lowering and anti‐hypertension medications, and hypertension status, and were stratified by T2D status and modelled at index ages of 40, 45, and 50 years.
**Figure S9:** Hazard ratios (HRs) for Mortality by BMI, T2D status, and age at index date after excluding hypertension, antihypertension use and lip‐lowering use covariates. The panels show the HRs for all‐cause mortality (Panel A), CVD mortality (Panel B), cancer mortality (Panel C), and mortality due to other causes (Panel D) in individuals with and without T2D across different BMI values compared to the reference group without T2D at a BMI of 25 kg/m^2^. Results are modelled at age at index date (40, 45, and 50 years). Shaded areas show 95% confidence intervals. HRs were adjusted for sex, ethnicity, deprivation, smoking status, and were stratified by T2D status and modelled at index ages of 40, 45, and 50 years.
**Figure S10:** Hazard ratios (HRs) for Mortality by BMI, T2D status, and age at index date among individuals without alcohol‐related conditions. The panels show the HRs for all‐cause mortality (Panel A), CVD mortality (Panel B), cancer mortality (Panel C), and mortality due to other causes (Panel D) in no alcohol‐related condition individuals with and without T2D across different BMI values compared to the reference group without T2D at a BMI of 25 kg/m^2^. Results are modelled at age at index date (40, 45, and 50 years). Shaded areas show 95% confidence intervals. HRs were adjusted for sex, ethnicity, deprivation, smoking status, use of lipid‐lowering and anti‐hypertension medications, and hypertension status, and were stratified by T2D status and modelled at index ages of 40, 45, and 50 years.
**Figure S11:** Hazard ratios (HRs) for Mortality by BMI, T2D status, and age at index date among non‐smokers. The panels show the HRs for all‐cause mortality (Panel A), CVD mortality (Panel B), cancer mortality (Panel C), and mortality due to other causes (Panel D) in non‐smokers with and without T2D across different BMI values compared to the reference group without T2D at a BMI of 25 kg/m^2^. Results are modelled at age at index date (40, 45, and 50 years). Shaded areas show 95% confidence intervals. HRs were adjusted for sex, ethnicity, deprivation, use of lipid‐lowering and anti‐hypertension medications, and hypertension status, and were stratified by T2D status and modelled at index ages of 40, 45, and 50 years.
**Figure S12:** Hazard ratios (HRs) for Mortality by BMI and age at index date among individuals with T2D, comparing complete case analysis and multiple imputation for missing data. The panels show the HRs for all‐cause mortality (Panel A), CVD mortality (Panel B), cancer mortality (Panel C), and mortality due to other causes (Panel D) in individuals with T2D across different BMI values compared to the reference group with T2D at a BMI of 25 kg/m^2^ by complete case analysis and multiple imputation. Results are modelled at age at index date (40, 45, and 50 years). Shaded areas show 95% confidence intervals. HRs were adjusted for sex, ethnicity, deprivation, smoking status, use of lipid‐lowering and anti‐hypertension medications, and hypertension status, and were stratified by T2D status and modelled at index ages of 40, 45, and 50 years.
**Table S1:** Other causes of deaths by T2D status.
**Table S2:** Other causes of deaths by T2D status among individuals with BMI < 25 kg/m2.
**Table S3:** Hazard Ratios (HRs) for mortality by T2D status across different BMI values among individuals with age 40, 45, and 50 at index date. The table shows the HRs with 95% confidence interval for all‐cause mortality, cancer mortality, CVD mortality, and other mortality in individuals with and without type 2 diabetes (T2D) at different BMI values of 20 kg/m2, 25 kg/m2, 30 kg/m2, 35 kg/m2, 40 kg/m2, 45 kg/m2, at index age 40, compared to the reference group without T2D at a BMI of 25 kg/m2.
**Table S4:** Ten‐year risks (cumulative incidences, %) of all‐cause mortality by T2D status across different BMI values among individuals with age 40, 45, and 50 years at index date.
**Table S5:** Ten‐year risks (i.e., cumulative incidences, %) of cause‐specific mortality by T2D status across different BMI values among individuals with age 40, 45, and 50 at index date. The table shows cumulative incidences of CVD mortality, cancer mortality, and mortality due to other causes in individuals with and without type 2 diabetes (T2D), at index ages of 40 across different BMI values of 20 kg/m^2^, 25 kg/m^2^, 30 kg/m^2^, 35 kg/m^2^, 40 kg/m^2^, 45 kg/m^2^.
**Table S6:** Ten‐year restricted mean survival time (RMST, years) and differences by type 2 diabetes status across BMI values among individuals aged 40, 45, and 50 years at index date.
**Table S7:** Sources of alcohol‐related clinical codes used in CPRD GOLD and Aurum.
**Table S8:** Baseline characteristics of individuals with and without recorded BMI, stratified by T2D status.

## Data Availability

The data that support the findings of this study were obtained from the Clinical Practice Research Datalink (CPRD) with linked Hospital Episode Statistics and Office for National Statistics mortality data. Restrictions apply to the availability of these data, which were used under licence for the current study and are not publicly available.
