# Supplementary material for: Body Mass Index and All‐Cause, Cancer, and Cardiovascular Mortality in Adults Aged 16–50 Years With and Without Type 2 Diabetes: An Analysis of Primary Care Records in England
Source: Diabetes Obes Metab. 2026 May 12;28(8):6687–98. doi: 10.1111/dom.70844 (PMC13341375; doi:10.1111/dom.70844)
Supplement: Supplementary file 1 — Figure S1: Study Population Selection Process Flow Chart. The overall selection process has been described previously (1). In the present study, only the additional selection criteria specific to this analysis are illustrated. Figure S2: Distribution of body mass index (BMI) in individuals with type 2 diabetes, comparing those with and without alcohol‐related disorders. Figure S3: Hazard Ratios (HRs) for mortality by BMI, T2D status, and age at index date. The panels show the HRs for all‐cause mortality (Panel A), CVD mortality (Panel B), cancer mortality (Panel C), mortality due to other causes (Panel D) in individuals with and without type 2 diabetes (T2D), at index ages of 40 and 50 years, across different BMI values compared to the reference group without T2D at a BMI of 25 kg/m2. Shaded areas show 95% confidence intervals. HRs were adjusted for sex, ethnicity, deprivation and smoking status, use of lipid‐lowering and anti‐hypertension medications, hypertension status, and were stratified by T2D status and modelled at index ages of 40 and 50 years. Figure S4: 10 year risks (cumulative incidence, %) of all‐cause mortality across BMI, stratified by age (40 and 50 years at index date) and T2D status. Shaded areas show 95% confidence intervals. 10‐year cumulative incidences were adjusted for sex, ethnicity, deprivation and smoking status, use of lipid‐lowering and anti‐hypertension medications, hypertension status, and were stratifiehd by T2D status and modelled at index ages of 40 and 50 years. Figure S5: 10‐year competing risk of cause‐specific mortality across BMI, stratified by T2D status. The panels show the 10‐year risks (cumulative incidences, %) of CVD mortality, cancer mortality, mortality due to other causes in individuals with and without T2D, modelled at index ages of 40 (Panel A) and 50 years (Panel B), across different BMI values. 10‐year cumulative incidences were adjusted for sex, ethnicity, deprivation and smoking status, use of lipid‐lowering and a [file DOM-28-6687-s001.docx]

**SUPPLEMENTARY MATERIAL**

**BODY MASS INDEX AND ALL-CAUSE, CANCER, AND CARDIOVASCULAR MORTALITY IN YOUNGER ADULTS WITH AND WITHOUT TYPE 2 DIABETES: AN ANALYSIS OF PRIMARY CARE RECORDS IN ENGLAND**


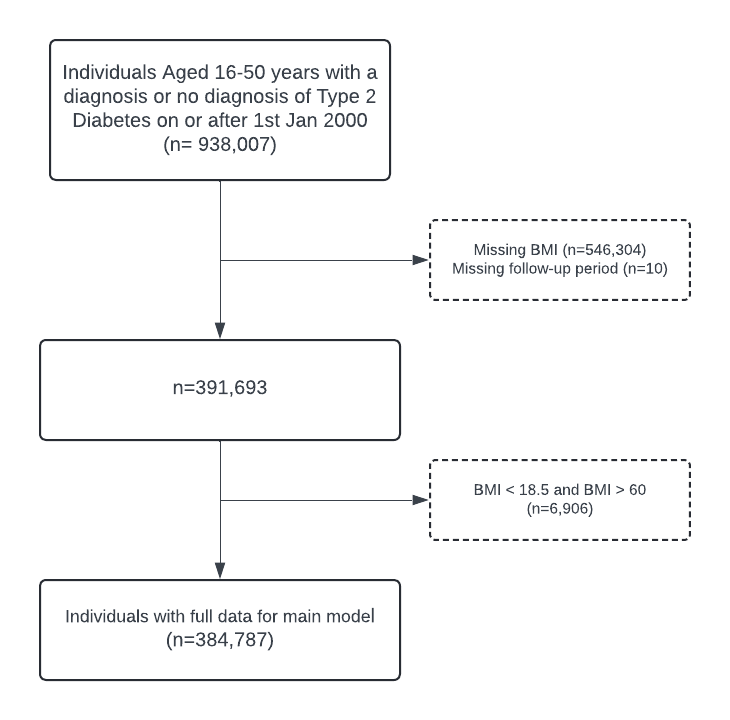


**Figure S1** – Study Population Selection Process Flow Chart. The overall selection process has been described previously (1).In the present study, only the additional selection criteria specific to this analysis are illustrated.

| **T2D** | | | **Non-T2D** | | |
| --- | --- | --- | --- | --- | --- |
| **Cause** | **Count** | **%** | **Cause** | **Count** | **%** |
| Alcoholic liver disease | 266 | 14.04 | Alcoholic liver disease | 666 | 16.35 |
| Pneumonia, organism unspecified | 118 | 6.23 | Other chronic obstructive pulmonary disease | 355 | 8.72 |
| Other chronic obstructive pulmonary disease | 115 | 6.07 | Pneumonia, organism unspecified | 192 | 4.71 |
| Unspecified diabetes mellitus | 98 | 5.17 | Accidental poisoning by and exposure to narcotics and psychodysleptics [hallucinogens], not elsewhere classified | 162 | 3.98 |
| Emergency use of U07 | 93 | 4.91 | Intentional self-harm by hanging, strangulation and suffocation | 139 | 3.41 |
| Fibrosis and cirrhosis of liver | 77 | 4.07 | Fibrosis and cirrhosis of liver | 119 | 2.92 |
| Type 2 diabetes mellitus | 65 | 3.43 | Emergency use of U07 | 106 | 2.60 |
| Obesity | 47 | 2.48 | Other ill-defined and unspecified causes of mortality | 82 | 2.01 |
| Other ill-defined and unspecified causes of mortality | 39 | 2.06 | Accidental poisoning by and exposure to other and unspecified drugs, medicaments and biological substances | 75 | 1.84 |
| Accidental poisoning by and exposure to narcotics and psychodysleptics [hallucinogens], not elsewhere classified | 38 | 2.01 | Mental and behavioural disorders due to use of alcohol | 70 | 1.72 |

**Table S1**  Other causes of deaths by T2D status

| **T2D** | | | **Non-T2D** | | |
| --- | --- | --- | --- | --- | --- |
| **Cause** | **Count** | **%** | **Cause** | **Count** | **%** |
| Alcoholic liver disease, unspecified | 19 | 7.36 | Alcoholic liver disease, unspecified | 134 | 7.45 |
| Alcoholic hepatic failure | 16 | 6.20 | Alcoholic cirrhosis of liver | 88 | 4.89 |
| Other and unspecified cirrhosis of liver | 12 | 4.65 | Alcoholic hepatic failure | 86 | 4.78 |
| Alcoholic cirrhosis of liver | 11 | 4.26 | Chronic obstructive pulmonary disease, unspecified | 81 | 4.51 |
| Pneumonia, unspecified | 11 | 4.26 | Chronic obstructive pulmonary disease with acute lower respiratory infection | 69 | 3.84 |
| Unspecified diabetes mellitus: With ketoacidosis | 10 | 3.88 | Other and unspecified cirrhosis of liver | 49 | 2.73 |
| COVID-19, virus identified | 10 | 3.88 | Other ill-defined and unspecified causes of mortality | 38 | 2.11 |
| Cystic fibrosis, unspecified | 9 | 3.49 | Pneumonia, unspecified | 30 | 1.67 |
| Chronic obstructive pulmonary disease with acute lower respiratory infection | 8 | 3.10 | Bronchopneumonia, unspecified | 29 | 1.61 |
| Hepatic failure, unspecified | 6 | 2.33 | Epilepsy, unspecified | 28 | 1.56 |
| Bronchopneumonia, unspecified | 6 | 2.33 | Multiple sclerosis | 28 | 1.56 |

**Table S2**  Other causes of deaths by T2D status among individuals with BMI <25 kg/m².

|  | | **T2D (95% Confidence Interval)** | | | | | **Non-T2D (95% Confidence Interval)** | | | | |
| --- | --- | --- | --- | --- | --- | --- | --- | --- | --- | --- | --- |
| Age (years) | BMI(kg/m²) | All-cause | CVD | Cancer | Other | All-cause | | CVD | Cancer | Other |  |
| 40 | 20 | 5.99 (4.61, 7.77) | 4.82 (2.31, 10.05) | 1.92 (0.91, 4.05) | 9.04 (6.67, 12.23) | 1.65 (1.47, 1.86) | | 1.37 (1.00, 1.88) | 1.49 (1.19, 1.85) | 1.84 (1.56, 2.16) |  |
|  | 25 | 2.12 (1.85, 2.43) | 2.33 (1.72, 3.14) | 1.23 (0.91, 1.67) | 2.64 (2.20, 3.17) | Ref (1) | | Ref (1) | Ref (1) | Ref (1) |  |
|  | 30 | 1.75 (1.57, 1.95) | 2.69 (2.11, 3.44) | 1.26 (1.00, 1.58) | 1.75 (1.51, 2.03) | 1.06 (1.00, 1.14) | | 1.43 (1.24, 1.65) | 1.09 (0.96, 1.24) | 0.94 (0.85, 1.03) |  |
|  | 35 | 1.94 (1.76, 2.15) | 3.84 (3.09, 4.78) | 1.44 (1.17, 1.77) | 1.68 (1.45, 1.94) | 1.37 (1.21, 1.55) | | 2.42 (1.85, 3.18) | 1.36 (1.07, 1.71) | 1.10 (0.92, 1.32) |  |
|  | 40 | 2.21 (1.98, 2.46) | 4.98 (3.97, 6.26) | 1.58 (1.26, 1.98) | 1.79 (1.54, 2.09) | 1.77 (1.53, 2.05) | | 3.85 (2.81, 5.28) | 1.62 (1.23, 2.12) | 1.35 (1.10, 1.66) |  |
|  | 45 | 2.55 (2.28, 2.85) | 5.99 (4.75, 7.55) | 1.68 (1.32, 2.14) | 2.09 (1.79, 2.45) | 2.30 (1.94, 2.73) | | 5.80 (4.14, 8.12) | 1.86 (1.29, 2.69) | 1.73 (1.35, 2.21) |  |
| 45 | 20 | 8.29 (6.61, 10.40) | 7.07 (4.14, 12.07) | 2.16 (1.16, 4.02) | 14.20 (10.81, 18.64) | 1.90 (1.72, 2.09) | | 1.54 (1.21, 1.97) | 1.56 (1.32, 1.84) | 2.28 (1.98, 2.62) |  |
|  | 25 | 2.03 (1.81, 2.26) | 2.17 (1.73, 2.72) | 1.52 (1.23, 1.87) | 2.33 (1.97, 2.75) | Ref (1) | | Ref (1) | Ref (1) | Ref (1) |  |
|  | 30 | 1.49 (1.36, 1.62) | 1.94 (1.62, 2.32) | 1.41 (1.20, 1.64) | 1.40 (1.23, 1.60) | 1.00 (0.95, 1.05) | | 1.37 (1.24, 1.51) | 1.01 (0.93, 1.10) | 0.84 (0.78, 0.91) |  |
|  | 35 | 1.63 (1.50, 1.77) | 2.44 (2.07, 2.88) | 1.47 (1.26, 1.70) | 1.42 (1.24, 1.62) | 1.24 (1.13, 1.36) | | 2.11 (1.75, 2.54) | 1.17 (1.00, 1.37) | 0.95 (0.82, 1.10) |  |
|  | 40 | 1.89 (1.72, 2.07) | 3.06 (2.56, 3.65) | 1.60 (1.36, 1.88) | 1.61 (1.39, 1.85) | 1.51 (1.36, 1.69) | | 2.79 (2.26, 3.46) | 1.34 (1.12, 1.61) | 1.16 (0.97, 1.37) |  |
|  | 45 | 2.28 (2.06, 2.51) | 3.80 (3.16, 4.58) | 1.80 (1.51, 2.16) | 1.99 (1.72, 2.31) | 1.84 (1.62, 2.10) | | 3.28 (2.55, 4.22) | 1.53 (1.21, 1.93) | 1.52 (1.24, 1.86) |  |
| 50 | 20 | 4.46 (3.12, 6.37) | 2.88 (1.15, 7.18) | 3.28 (1.69, 6.35) | 7.40 (4.60, 11.91) | 1.88 (1.66, 2.13) | | 1.08 (0.77, 1.51) | 1.39 (1.13, 1.71) | 2.98 (2.48, 3.59) |  |
|  | 25 | 1.63 (1.41, 1.90) | 1.31 (0.96, 1.79) | 1.56 (1.22, 1.99) | 2.03 (1.60, 2.57) | Ref (1) | | Ref (1) | Ref (1) | Ref (1) |  |
|  | 30 | 1.29 (1.15, 1.45) | 1.35 (1.06, 1.70) | 1.25 (1.04, 1.50) | 1.38 (1.15, 1.66) | 0.97 (0.91, 1.03) | | 1.08 (0.95, 1.23) | 0.93 (0.84, 1.03) | 1.00 (0.90, 1.10) |  |
|  | 35 | 1.39 (1.24, 1.55) | 1.78 (1.43, 2.21) | 1.23 (1.02, 1.48) | 1.42 (1.18, 1.70) | 1.18 (1.05, 1.32) | | 1.31 (1.04, 1.66) | 0.99 (0.82, 1.19) | 1.37 (1.14, 1.64) |  |
|  | 40 | 1.61 (1.42, 1.82) | 2.30 (1.82, 2.91) | 1.26 (1.03, 1.56) | 1.68 (1.37, 2.06) | 1.46 (1.28, 1.66) | | 1.70 (1.29, 2.24) | 1.12 (0.91, 1.40) | 1.80 (1.46, 2.22) |  |
|  | 45 | 1.97 (1.73, 2.25) | 2.92 (2.27, 3.75) | 1.35 (1.06, 1.72) | 2.23 (1.81, 2.75) | 1.84 (1.57, 2.16) | | 2.34 (1.71, 3.21) | 1.35 (1.02, 1.79) | 2.29 (1.78, 2.94) |  |

**Table S3** -Hazard ratios (HRs) for mortality by T2D status across different BMI values among individuals with age 40, 45, and 50 at index date. The table shows the HRs with 95% confidence interval for all-cause mortality, cancer mortality, CVD mortality, and other mortality in individuals with and without type 2 diabetes (T2D) at different BMI values of 20 kg/m², 25 kg/m², 30 kg/m², 35 kg/m², 40 kg/m², 45 kg/m², at index age 40, compared to the reference group without T2D at a BMI of 25 kg/m².

| **Age (years)** | **BMI (kg/m^2^)** | **T2D (95% Confidence Interval)** | **Non T2D (95% Confidence Interval)** |
| --- | --- | --- | --- |
| 40 | 20 | 8.76 (6.66, 10.81) | 2.53 (2.29, 2.77) |
|  | 25 | 3.23 (2.85, 3.60) | 1.54 (1.44, 1.64) |
|  | 30 | 2.68 (2.45, 2.90) | 1.64 (1.53, 1.74) |
|  | 35 | 2.97 (2.74, 3.19) | 2.10 (1.91, 2.30) |
|  | 40 | 3.36 (3.08, 3.64) | 2.71 (2.41, 3.00) |
|  | 45 | 3.86 (3.53, 4.19) | 3.50 (2.99, 4.00) |
| 45 | 20 | 17.33 (13.91, 20.62) | 4.38 (4.03, 4.73) |
|  | 25 | 4.66 (4.21, 5.11) | 2.34 (2.22, 2.46) |
|  | 30 | 3.45 (3.21, 3.69) | 2.35 (2.24, 2.46) |
|  | 35 | 3.78 (3.53, 4.03) | 2.88 (2.69, 3.08) |
|  | 40 | 4.35 (4.03, 4.67) | 3.52 (3.24, 3.79) |
|  | 45 | 5.22 (4.80, 5.63) | 4.26 (3.78, 4.73) |
| 50 | 20 | 14.66 (9.91, 19.15) | 6.57 (5.89, 7.25) |
|  | 25 | 5.75 (5.00, 6.49) | 3.57 (3.35, 3.79) |
|  | 30 | 4.58 (4.15, 5.00) | 3.48 (3.28, 3.67) |
|  | 35 | 4.92 (4.48, 5.36) | 4.18 (3.84, 4.52) |
|  | 40 | 5.67 (5.09, 6.24) | 5.15 (4.65, 5.64) |
|  | 45 | 6.89 (6.13, 7.64) | 6.44 (5.57, 7.30) |

**Table S4** –10-year risks (cumulative incidences, %) of all-cause mortality by T2D status across different BMI values among individuals with age 40, 45, and 50 years at index date.

**Table S5**– 10-year risks (i.e., cumulative incidences, %) of cause-specific mortality by T2D status across different BMI values among individuals with age 40, 45, and 50 at index date. The table shows cumulative incidences of CVD mortality, cancer mortality, and mortality due to other causes in individuals with and without type 2 diabetes (T2D), at index ages of 40 across different BMI values of 20 kg/m², 25 kg/m², 30 kg/m², 35 kg/m², 40 kg/m², 45 kg/m².

|  | | **T2D** | | | **Non-T2D** | | |
| --- | --- | --- | --- | --- | --- | --- | --- |
| **Age (years)** | **BMI (kg/m^2^)** | **CVD** | **Cancer** | **Other** | **CVD** | **Cancer** | **Other** |
| 40 | 20 | 1.00 | 0.88 | 7.24 | 0.30 | 0.72 | 1.55 |
|  | 25 | 0.51 | 0.59 | 2.22 | 0.22 | 0.49 | 0.85 |
|  | 30 | 0.59 | 0.61 | 1.48 | 0.32 | 0.53 | 0.80 |
|  | 35 | 0.84 | 0.69 | 1.41 | 0.54 | 0.66 | 0.93 |
|  | 40 | 1.09 | 0.76 | 1.51 | 0.85 | 0.78 | 1.14 |
|  | 45 | 1.31 | 0.80 | 1.75 | 1.27 | 0.89 | 1.45 |
| 45 | 20 | 2.56 | 1.58 | 13.77 | 0.63 | 1.28 | 2.48 |
|  | 25 | 0.89 | 1.24 | 2.53 | 0.42 | 0.83 | 1.10 |
|  | 30 | 0.80 | 1.16 | 1.53 | 0.57 | 0.84 | 0.93 |
|  | 35 | 1.01 | 1.21 | 1.55 | 0.87 | 0.97 | 1.04 |
|  | 40 | 1.25 | 1.31 | 1.75 | 1.15 | 1.11 | 1.27 |
|  | 45 | 1.55 | 1.47 | 2.16 | 1.34 | 1.25 | 1.65 |
| 50 | 20 | 1.79 | 4.74 | 8.72 | 0.72 | 2.15 | 3.75 |
|  | 25 | 0.88 | 2.42 | 2.57 | 0.69 | 1.58 | 1.29 |
|  | 30 | 0.92 | 1.95 | 1.76 | 0.75 | 1.46 | 1.28 |
|  | 35 | 1.21 | 1.92 | 1.81 | 0.90 | 1.55 | 1.75 |
|  | 40 | 1.55 | 1.97 | 2.14 | 1.16 | 1.75 | 2.29 |
|  | 45 | 1.95 | 2.08 | 2.81 | 1.57 | 2.09 | 2.89 |

| **Age (years)** | **BMI (kg/m^2^)** | **T2D (95% Confidence Interval)** | **Non-T2D (95% Confidence Interval)** | **Difference (95% Confidence Interval)** |
| --- | --- | --- | --- | --- |
| 40 | 20 | 9.63 (9.53, 9.72) | 9.89 (9.88, 9.90) | -0.27 (-0.36, -0.18) |
|  | 25 | 9.86 (9.85, 9.88) | 9.94 (9.93, 9.94) | -0.07 (-0.09, -0.05) |
|  | 30 | 9.89 (9.88, 9.90) | 9.93 (9.93, 9.94) | -0.04 (-0.05, -0.03) |
|  | 35 | 9.88 (9.87, 9.88) | 9.91 (9.90, 9.92) | -0.04 (-0.05, -0.02) |
|  | 40 | 9.86 (9.85, 9.87) | 9.89 (9.87, 9.90) | -0.03 (-0.04, -0.01) |
|  | 45 | 9.84 (9.82, 9.85) | 9.85 (9.83, 9.87) | -0.02 (-0.04, 0.01) |
| 45 | 20 | 9.24 (9.09, 9.40) | 9.81 (9.80, 9.83) | -0.57 (-0.73, -0.42) |
|  | 25 | 9.80 (9.78, 9.82) | 9.90 (9.90, 9.91) | -0.10 (-0.12, -0.08) |
|  | 30 | 9.85 (9.84, 9.87) | 9.90 (9.90, 9.91) | -0.05 (-0.06, -0.04) |
|  | 35 | 9.84 (9.83, 9.85) | 9.88 (9.87, 9.89) | -0.04 (-0.05, -0.02) |
|  | 40 | 9.82 (9.80, 9.83) | 9.85 (9.84, 9.86) | -0.04 (-0.05, -0.02) |
|  | 45 | 9.78 (9.76, 9.80) | 9.82 (9.80, 9.84) | -0.04 (-0.07, -0.01) |
| 50 | 20 | 9.36 (9.16, 9.57) | 9.72 (9.69, 9.75) | -0.36 (-0.57, -0.15) |
|  | 25 | 9.76 (9.72, 9.79) | 9.85 (9.84, 9.86) | -0.09 (-0.13, -0.06) |
|  | 30 | 9.81 (9.79, 9.82) | 9.85 (9.85, 9.86) | -0.05 (-0.07, -0.03) |
|  | 35 | 9.79 (9.77, 9.81) | 9.82 (9.81, 9.84) | -0.03 (-0.05, -0.01) |
|  | 40 | 9.76 (9.74, 9.78) | 9.78 (9.76, 9.80) | -0.02 (-0.05, 0.01) |
|  | 45 | 9.71 (9.67, 9.74) | 9.73 (9.69, 9.76) | -0.02 (-0.07, 0.03) |

**Table S6** –Ten-year restricted mean survival time (RMST, years) and differences by type 2 diabetes status across BMI values among individuals aged 40, 45, and 50 years at index date.

| **Database** | **Code Group Name** | **Code Type** | **Source URL** |
| --- | --- | --- | --- |
| CPRD GOLD | res41-alcohol | Read | <https://clinicalcodes.rss.mhs.man.ac.uk/medcodes/article/41/codelist/res41-alcohol/> |
|  | res47-alcohol-intake |  | <https://clinicalcodes.rss.mhs.man.ac.uk/medcodes/article/47/codelist/res47-alcohol-intake/> |
| CPRD Aurum | ALC_COD | SNOMED | <https://www.opencodelists.org/> |
|  | ALCUSAGE_COD |  |  |
|  | EXCESSALC_COD |  |  |
|  | AUDIT_COD |  |  |
|  | AUDITC_COD |  |  |
|  | EXCESSALC_COD |  |  |
|  | Multimorbidity_alcoholproblems |  |  |
|  | alcohol_problems |  |  |
|  | ALCADV_COD |  |  |
|  | ALCOHOLINT_COD |  |  |
|  | ALCBRINT_COD |  |  |
|  | ALCEXINT_COD |  |  |
|  | ALCINTDEC_COD |  |  |
|  | ALCBRINTDEC_COD |  |  |
|  | ALCEXINTDEC_COD |  |  |
|  | ALCEX_COD, |  |  |
|  | ALCSCRNDEC_COD |  |  |
|  | ALCREF_COD |  |  |
|  | ALCSPADV_COD |  |  |
|  | ALCSPADVDEC_COD |  |  |
|  | FAST_COD |  |  |
|  | Hazardous drinking |  |  |

**Table S7**– Sources of alcohol-related clinical codes used in CPRD GOLD and Aurum

|  | **T2D** | | **Non-T2D** | |
| --- | --- | --- | --- | --- |
|  | **Without BMI**  n=21,455 | **With BMI**  n=86,606 | **Without BMI**  n=531,765 | **With BMI**  n=298,181 |
| **Follow-up time (years)** | | | | |
| mean (SD) | 11.49 (6.15) | 9.06 (5.46) | 9.65 (5.88) | 10.02 (5.56) |
| **Age at index** | | | | |
| mean (SD) | 42.33 (6.55) | 42.45 (6.44) | 42.14 (6.55) | 42.43 (6.63) |
| **Sex (%)** | | | | |
| Male | 13233 (61.7) | 45520 (52.6) | 296481 (55.8) | 142553 (47.8) |
| **Ethnicity (%)** | | | | |
| White | 15560 (72.5) | 58111 (67.1) | 437536 (82.3) | 235054 (78.8) |
| Black | 1364 (6.4) | 7261 (8.4) | 28510 (5.4) | 18134 (6.1) |
| South Asian | 2847 (13.3) | 13551 (15.6) | 33727 (6.3) | 24968 (8.4) |
| Other | 1684 (7.8) | 7683 (8.9) | 31992 (6.0) | 20025 (6.7) |
| **IMD** | | | | |
| 1 (Most deprived) | 2890 (13.5) | 9442 (10.9) | 77338 (14.5) | 42535 (14.3) |
| 2 | 3036 (14.2) | 11379 (13.1) | 82258 (15.5) | 46328 (15.5) |
| 3 | 3794 (17.7) | 14737 (17.0) | 95659 (18.0) | 53207 (17.8) |
| 4 | 5230 (24.4) | 21510 (24.8) | 123152 (23.2) | 68268 (22.9) |
| 5 | 6505 (30.3) | 29538 (34.1) | 153358 (28.8) | 87843 (29.5) |
| **Smoke** | | | | |
| Yes (%) | 3221 (15.0) | 22476 (26.0) | 31100 (5.8) | 72766 (24.4) |
| **Lipid-lowering medication** | | | | |
| Yes (%) | 2266 (10.6) | 17748 (20.5) | 2780 (0.5) | 13299 (4.5) |
| **Antihypertensive medication** | | | | |
| Yes (%) | 4532 (21.1) | 27451 (31.7) | 11184 (2.1) | 32334 (10.8) |
| **Hypertension** | | | | |
| Yes (%) | 590 (2.7) | 2375 (2.7) | 2284 (0.4) | 3427 (1.1) |

**Table S8**– Baseline characteristics of individuals with and without recorded BMI, stratified by T2D status


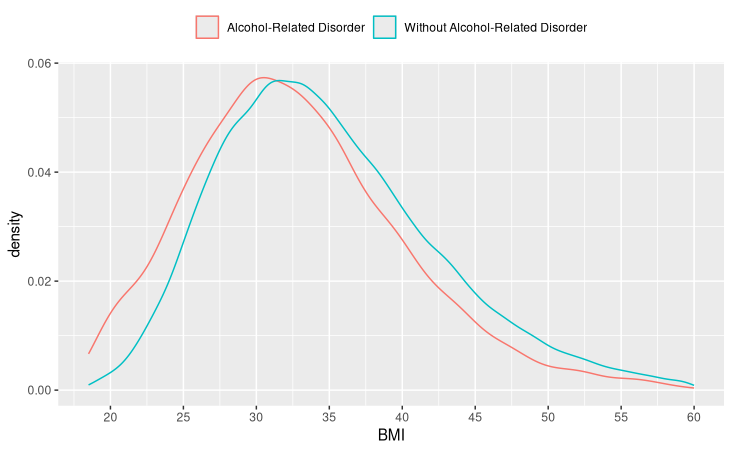


**Figure S2**—Distribution of body mass index (BMI) in individuals with type 2 diabetes, comparing those with and without alcohol-related disorders.


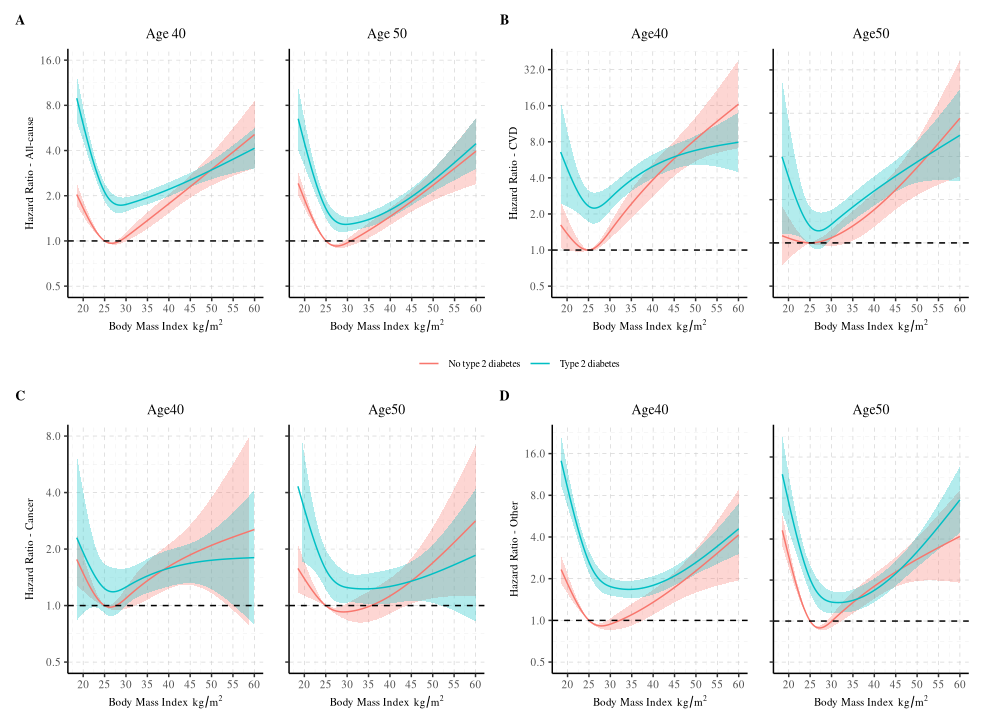


**Figure S3**—Hazard ratios (HRs) for mortality by BMI, T2D status, and age at index date. The panels show the HRs for all-cause mortality (Panel A), CVD mortality (Panel B), cancer mortality (Panel C), mortality due to other causes (Panel D) in individuals with and without type 2 diabetes (T2D), at index ages of 40 and 50 years, across different BMI values compared to the reference group without T2D at a BMI of 25 kg/m². Shaded areas show 95% confidence intervals. HRs were adjusted for sex, ethnicity, deprivation and smoking status, use of lipid-lowering and anti-hypertension medications, hypertension status, and were stratified by T2D status and modelled at index ages of 40 and 50 years.


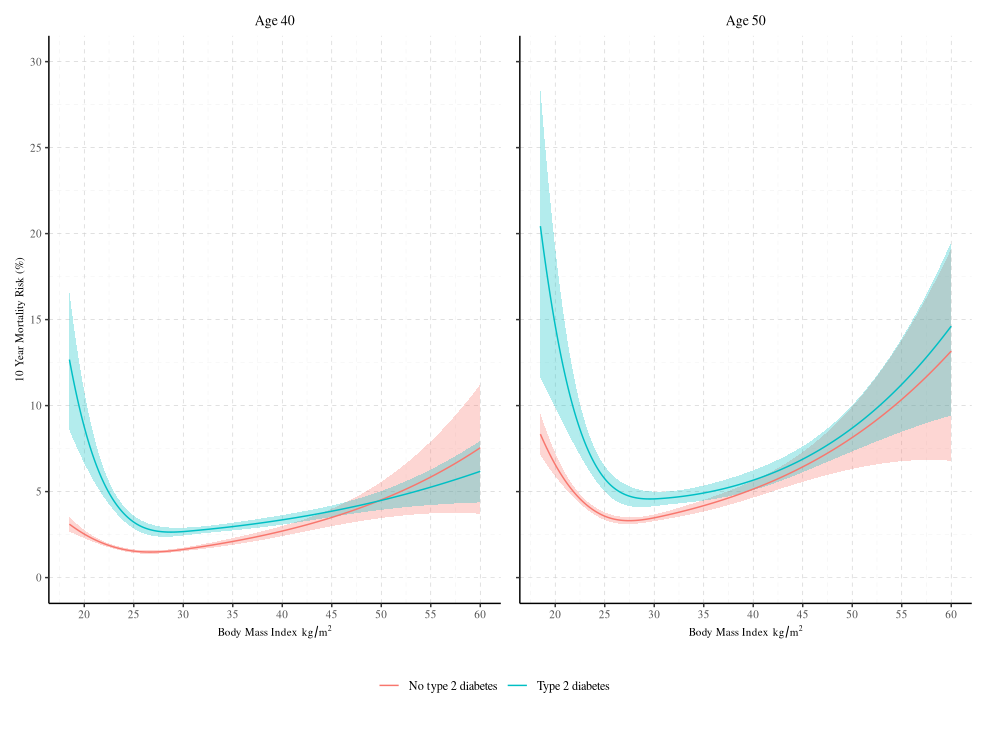


**Figure S4**—10–year risks (cumulative incidence, %) of all-cause mortality across BMI, stratified by age (40 and 50 years at index date) and T2D status. Shaded areas show 95% confidence intervals. 10-year cumulative incidences were adjusted for sex, ethnicity, deprivation and smoking status, use of lipid-lowering and anti-hypertension medications, hypertension status, and were stratified by T2D status and modelled at index ages of 40 and 50 years.


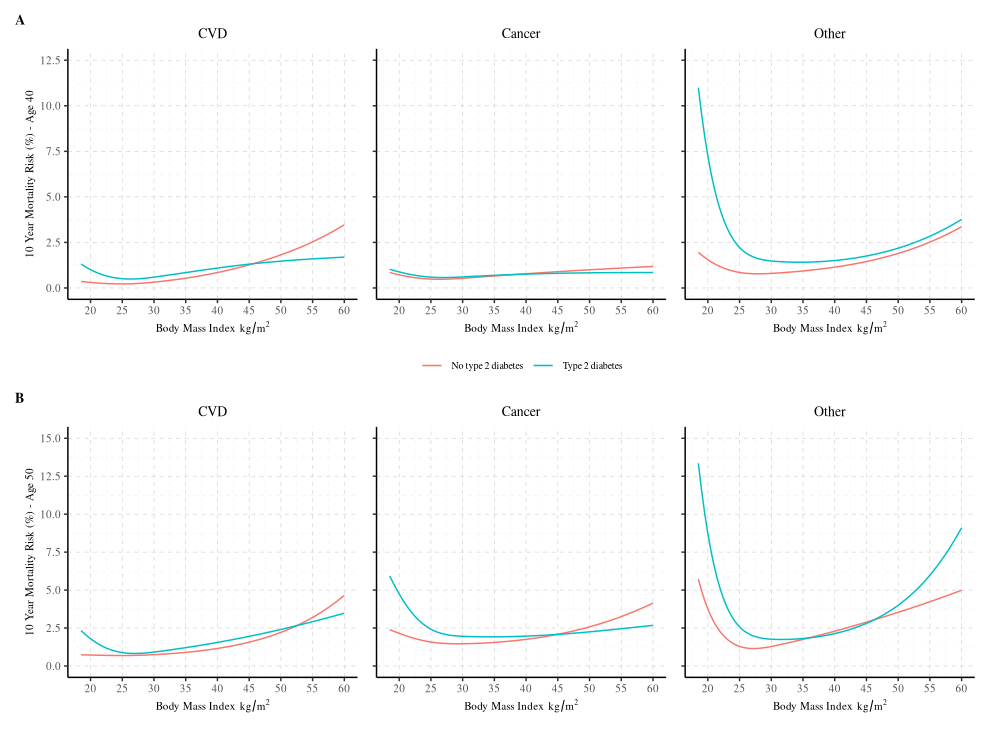


**Figure S5**—10-year competing risk of cause-specific mortality across BMI, stratified by T2D status. The panels show the 10-year risks (cumulative incidences, %) of CVD mortality, cancer mortality, mortality due to other causes in individuals with and without T2D, modelled at index ages of 40 (Panel A) and 50 years (Panel B), across different BMI values. 10-year cumulative incidences were adjusted for sex, ethnicity, deprivation and smoking status, use of lipid-lowering and anti-hypertension medications, and hypertension status, and were stratified by T2D status and modelled at index ages of 40 and 50 years.


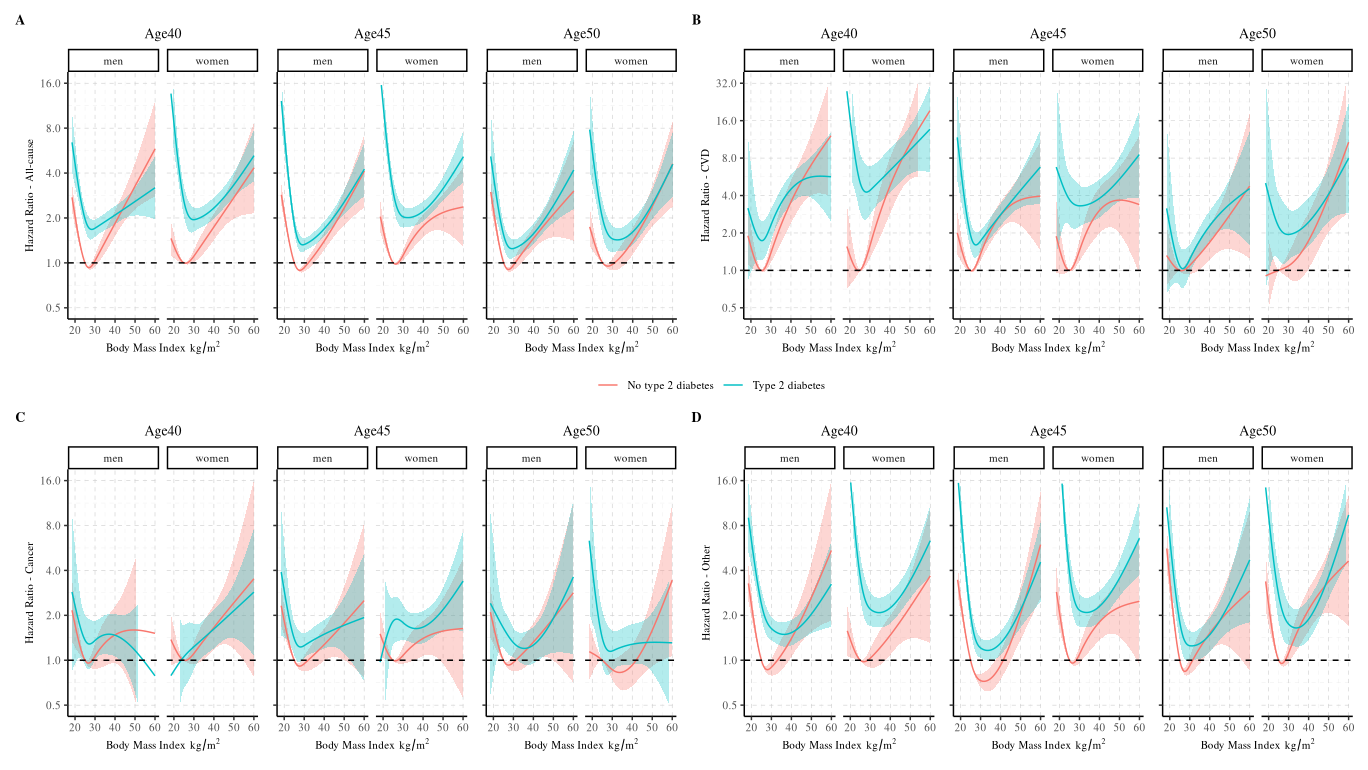


**Figure S6**—Hazard ratios (HRs) for mortality by BMI, T2D status, and age at index date among male and female subgroups. The panels show the HRs for all-cause mortality (Panel A), CVD mortality (Panel B), cancer mortality (Panel C), and mortality due to other causes (Panel D) with and without T2D across different BMI values compared to the reference group without T2D at a BMI of 25 kg/m². Results are presented separately for males and females and further modelled at age at index date (40, 45, and 50 years). Shaded areas show 95% confidence intervals. HRs were adjusted for sex, ethnicity, deprivation, smoking status, use of lipid-lowering and anti-hypertension medications, and hypertension status, and were stratified by T2D status and modelled at index ages of 40, 45, and 50 years.


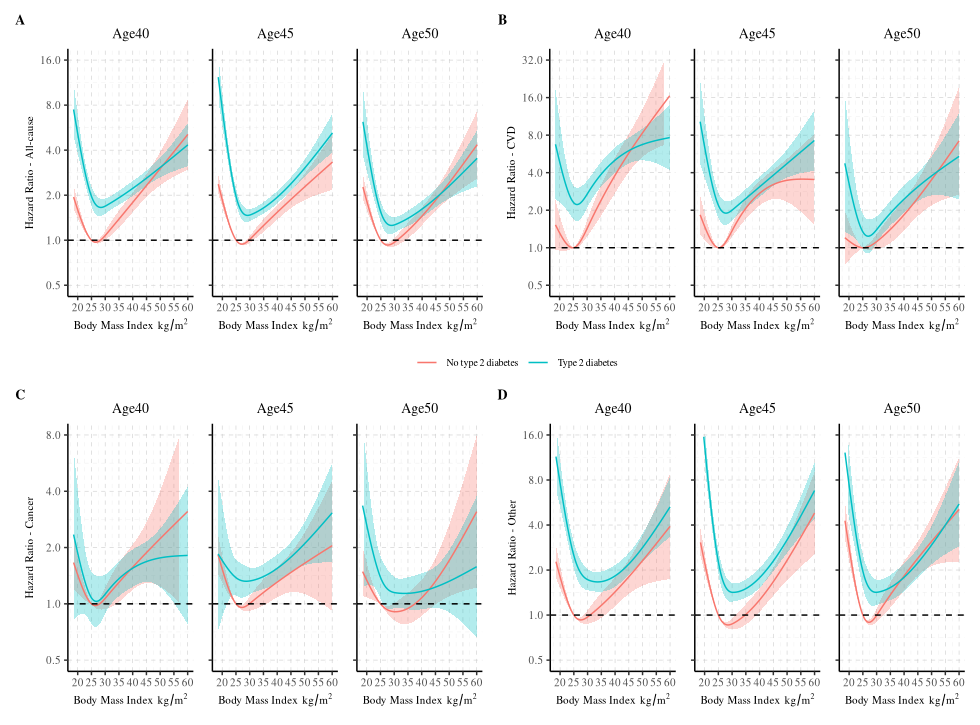


**Figure S7**—Hazard Ratios (HRs) for Mortality by BMI, T2D status, and age at index date after excluding mortality cases less than 2-year follow-up. The panels show the HRs for all-cause mortality (Panel A), CVD mortality (Panel B), cancer mortality (Panel C), and mortality due to other causes (Panel D) in individuals alive more than 2 follow-up years with and without T2D across different BMI values compared to the reference group without T2D at a BMI of 25 kg/m². Results are modelled at age at index date (40, 45, and 50 years). Shaded areas show 95% confidence intervals. HRs were adjusted for sex, ethnicity, deprivation, smoking status, use of lipid-lowering and anti-hypertension medications, and hypertension status, and were stratified by T2D status and modelled at index ages of 40, 45, and 50 years.


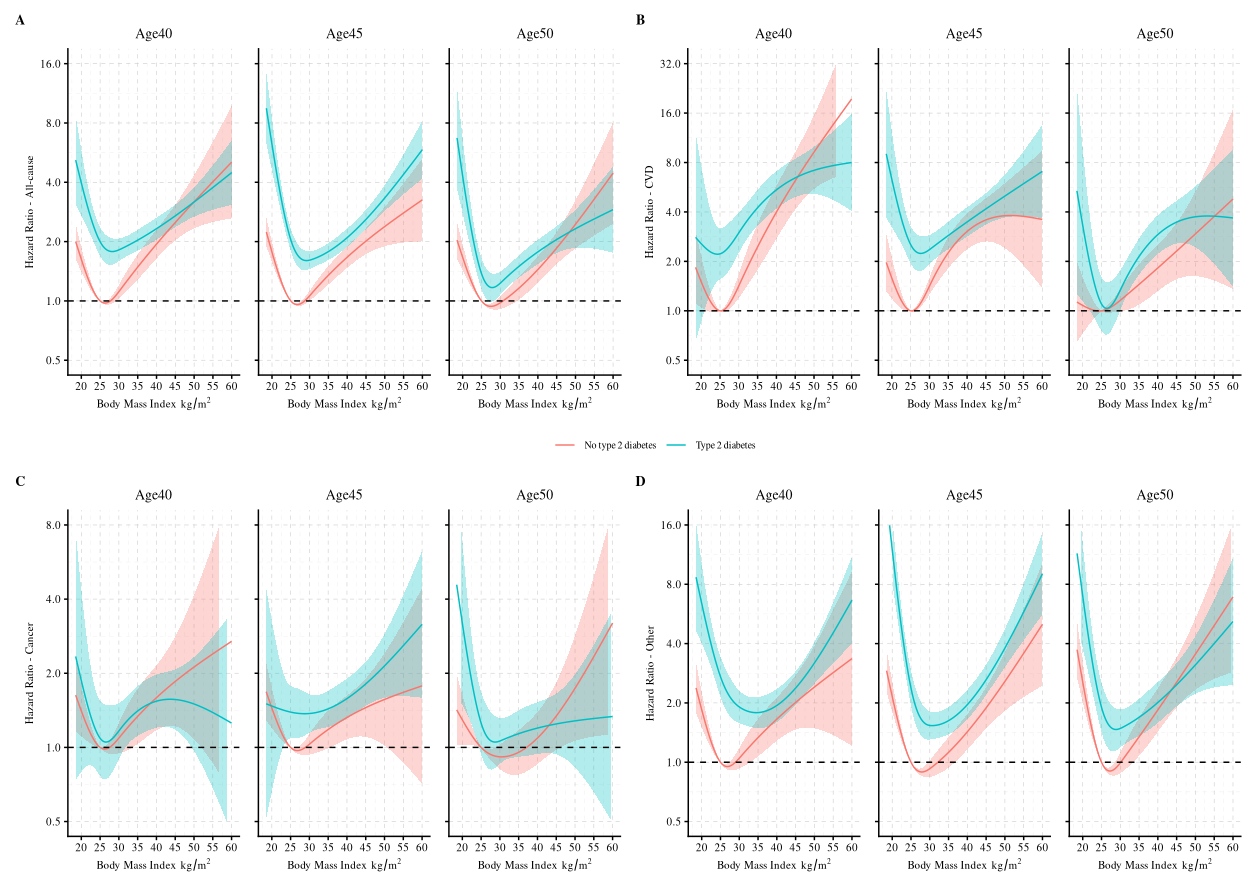


**Figure S8**—Hazard Ratios (HRs) for Mortality by BMI, T2D status, and age at index date after excluding mortality cases less than 5-year follow-up. The panels show the HRs for all-cause mortality (Panel A), CVD mortality (Panel B), cancer mortality (Panel C), and mortality due to other causes (Panel D) in individuals alive more than 5 follow-up years with and without T2D across different BMI values compared to the reference group without T2D at a BMI of 25 kg/m². Results are modelled at age at index date (40, 45, and 50 years). Shaded areas show 95% confidence intervals. HRs were adjusted for sex, ethnicity, deprivation, smoking status, use of lipid-lowering and anti-hypertension medications, and hypertension status, and were stratified by T2D status and modelled at index ages of 40, 45, and 50 years.


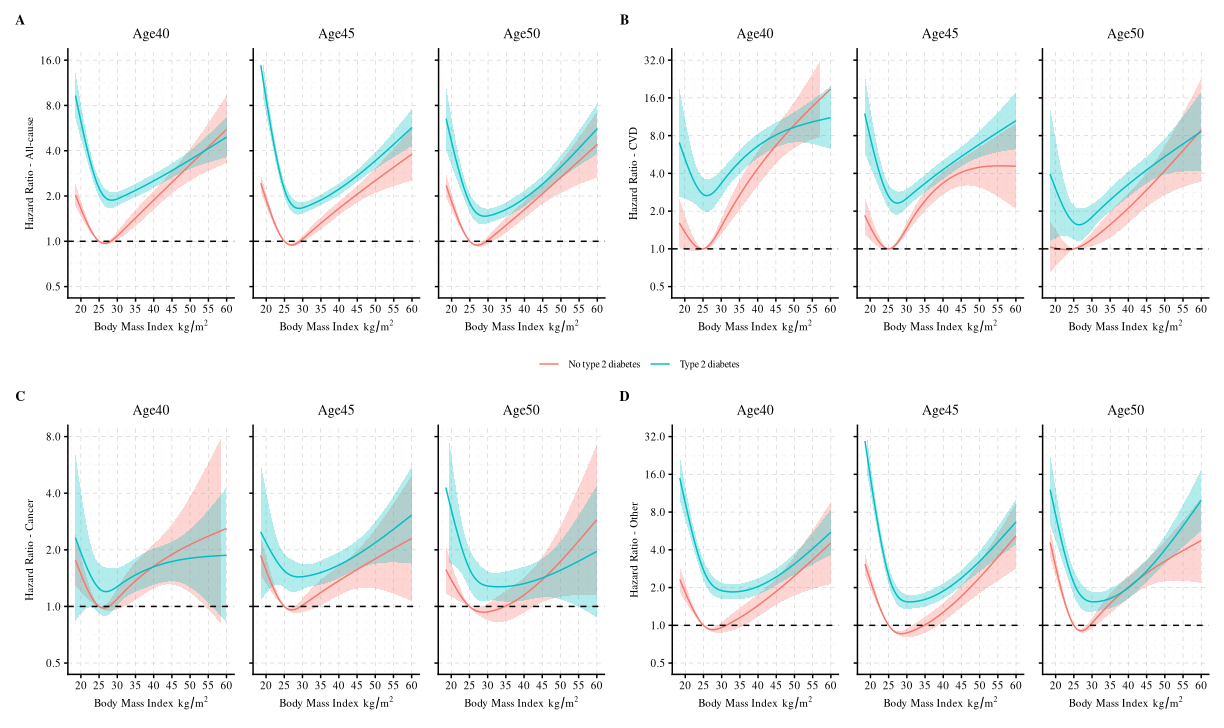


**Figure S9**—Hazard Ratios (HRs) for Mortality by BMI, T2D status, and age at index date after excluding hypertension, antihypertension use and lip-lowering use covariates. The panels show the HRs for all-cause mortality (Panel A), CVD mortality (Panel B), cancer mortality (Panel C), and mortality due to other causes (Panel D) in individuals with and without T2D across different BMI values compared to the reference group without T2D at a BMI of 25 kg/m². Results are modelled at age at index date (40, 45, and 50 years). Shaded areas show 95% confidence intervals. HRs were adjusted for sex, ethnicity, deprivation, smoking status, and were stratified by T2D status and modelled at index ages of 40, 45, and 50 years.


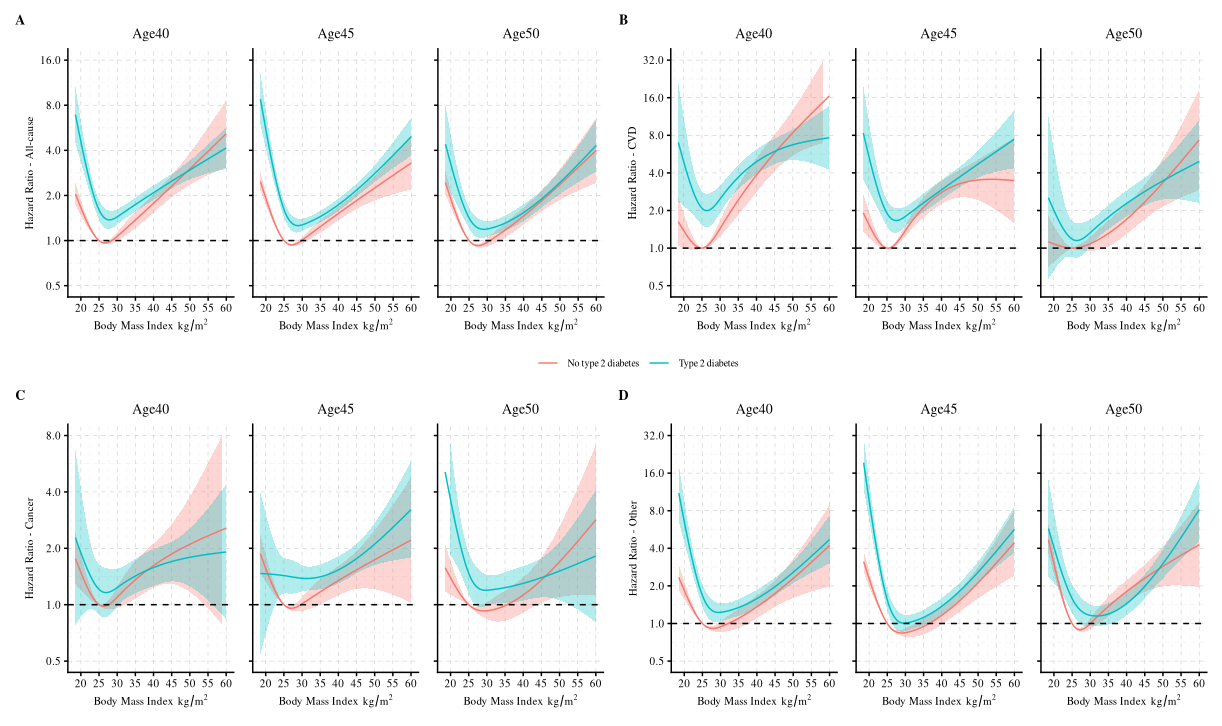


**Figure S10**—Hazard Ratios (HRs) for Mortality by BMI, T2D status, and age at index date among individuals without alcohol-related condition. The panels show the HRs for all-cause mortality (Panel A), CVD mortality (Panel B), cancer mortality (Panel C), and mortality due to other causes (Panel D) in no alcohol-related condition individuals with and without T2D across different BMI values compared to the reference group without T2D at a BMI of 25 kg/m². Results are modelled at age at index date (40, 45, and 50 years). Shaded areas show 95% confidence intervals. HRs were adjusted for sex, ethnicity, deprivation, smoking status, use of lipid-lowering and anti-hypertension medications, and hypertension status, and were stratified by T2D status and modelled at index ages of 40, 45, and 50 years.


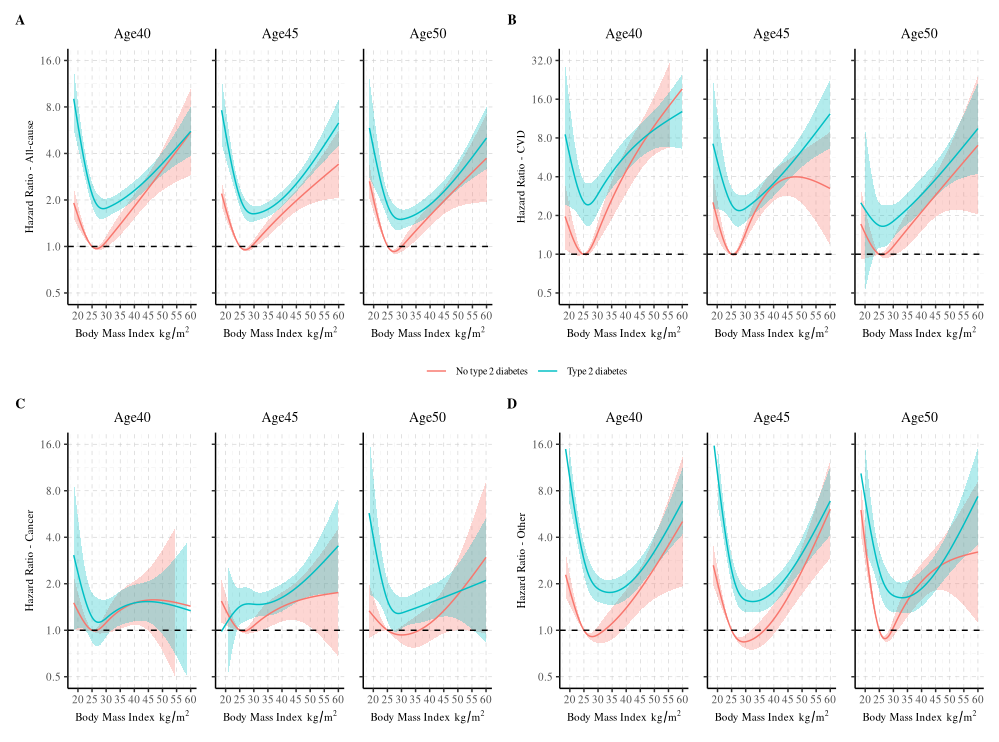


**Figure S11**—Hazard Ratios (HRs) for Mortality by BMI, T2D status, and age at index date among non-smokers. The panels show the HRs for all-cause mortality (Panel A), CVD mortality (Panel B), cancer mortality (Panel C), and mortality due to other causes (Panel D) in non-smokers with and without T2D across different BMI values compared to the reference group without T2D at a BMI of 25 kg/m². Results are modelled at age at index date (40, 45, and 50 years). Shaded areas show 95% confidence intervals. HRs were adjusted for sex, ethnicity, deprivation, use of lipid-lowering and anti-hypertension medications, and hypertension status, and were stratified by T2D status and modelled at index ages of 40, 45, and 50 years.


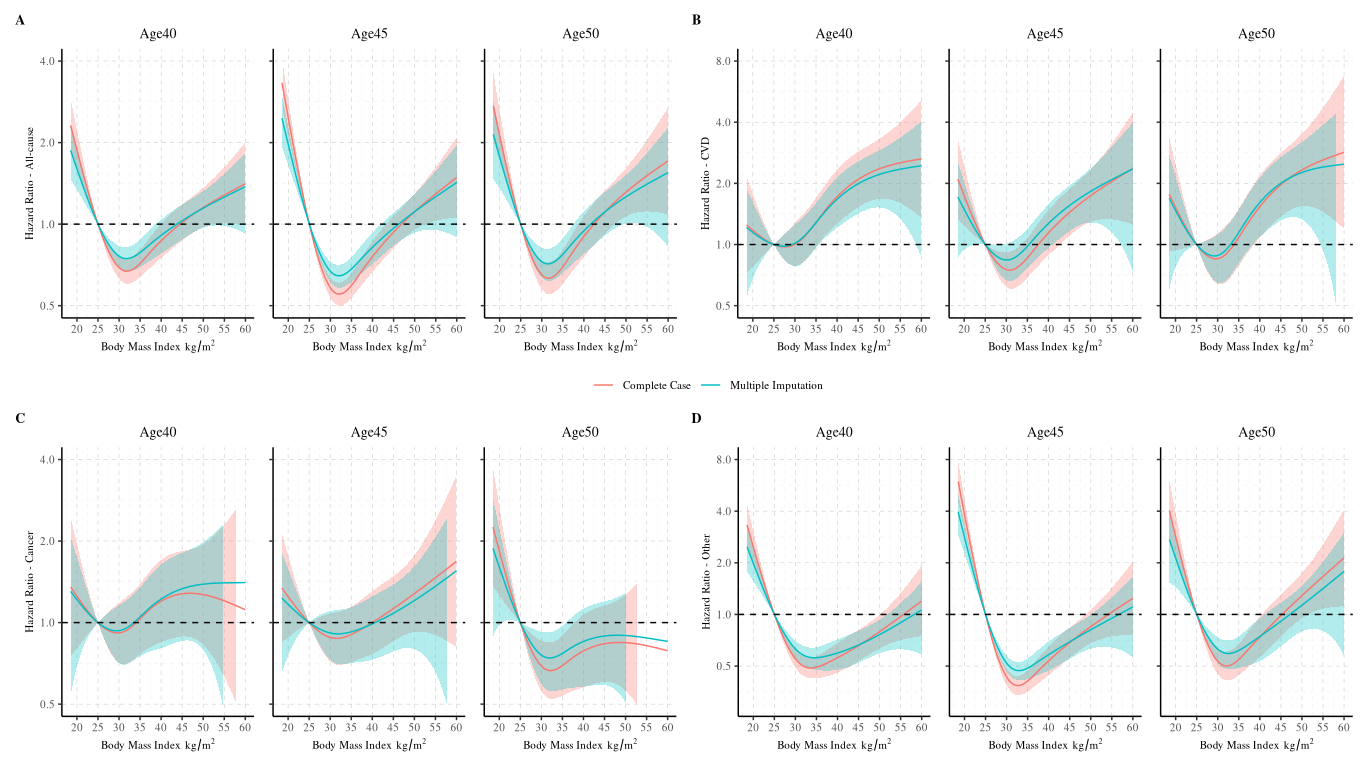


**Figure S12**—Hazard Ratios (HRs) for Mortality by BMI and age at index date among individuals with T2D, comparing complete case analysis and multiple imputation for missing data. The panels show the HRs for all-cause mortality (Panel A), CVD mortality (Panel B), cancer mortality (Panel C), and mortality due to other causes (Panel D) in individuals with T2D across different BMI values compared to the reference group with T2D at a BMI of 25 kg/m² by complete case analysis and multiple imputation. Results are modelled at age at index date (40, 45, and 50 years). Shaded areas show 95% confidence intervals. HRs were adjusted for sex, ethnicity, deprivation, smoking status, use of lipid-lowering and anti-hypertension medications, and hypertension status, and were stratified by T2D status and modelled at index ages of 40, 45, and 50 years.

**RECORD checklist**

|  | **Item No.** | **STROBE items** | **Location in manuscript where items are reported** | **RECORD items** | **Location in manuscript where items are reported** |
| --- | --- | --- | --- | --- | --- |
| **Title and abstract** | | | | | |
|  | 1 | (a) Indicate the study’s design with a commonly used term in the title or the abstract (b) Provide in the abstract an informative and balanced summary of what was done and what was found | Pages 1-2 | RECORD 1.1: The type of data used should be specified in the title or abstract. When possible, the name of the databases used should be included.  RECORD 1.2: If applicable, the geographic region and timeframe within which the study took place should be reported in the title or abstract.  RECORD 1.3: If linkage between databases was conducted for the study, this should be clearly stated in the title or abstract. | Pages 1-2 |
| **Introduction** | | | | | |
| Background rationale | 2 | Explain the scientific background and rationale for the investigation being reported | Pages 3-4 |  |  |
| Objectives | 3 | State specific objectives, including any prespecified hypotheses | Pages 3-4 |  |  |
| **Methods** | | | | | |
| Study Design | 4 | Present key elements of study design early in the paper | Page 4 |  |  |
| Setting | 5 | Describe the setting, locations, and relevant dates, including periods of recruitment, exposure, follow-up, and data collection | Pages 4-5 |  |  |
| Participants | 6 | *(a) Cohort study* - Give the eligibility criteria, and the sources and methods of selection of participants. Describe methods of follow-up  *Case-control study* - Give the eligibility criteria, and the sources and methods of case ascertainment and control selection. Give the rationale for the choice of cases and controls  *Cross-sectional study* - Give the eligibility criteria, and the sources and methods of selection of participants  *(b) Cohort study* - For matched studies, give matching criteria and number of exposed and unexposed  *Case-control study* - For matched studies, give matching criteria and the number of controls per case | Page 5 | RECORD 6.1: The methods of study population selection (such as codes or algorithms used to identify subjects) should be listed in detail. If this is not possible, an explanation should be provided.  RECORD 6.2: Any validation studies of the codes or algorithms used to select the population should be referenced. If validation was conducted for this study and not published elsewhere, detailed methods and results should be provided.  RECORD 6.3: If the study involved linkage of databases, consider use of a flow diagram or other graphical display to demonstrate the data linkage process, including the number of individuals with linked data at each stage. | Appendix Figure S1 |
| Variables | 7 | Clearly define all outcomes, exposures, predictors, potential confounders, and effect modifiers. Give diagnostic criteria, if applicable. | Pages 5-6 | RECORD 7.1: A complete list of codes and algorithms used to classify exposures, outcomes, confounders, and effect modifiers should be provided. If these cannot be reported, an explanation should be provided. | codes are in the code files in online repository |
| Data sources/ measurement | 8 | For each variable of interest, give sources of data and details of methods of assessment (measurement).  Describe comparability of assessment methods if there is more than one group | Pages 4-5 |  |  |
| Bias | 9 | Describe any efforts to address potential sources of bias |  |  |  |
| Study size | 10 | Explain how the study size was arrived at | Page 5 |  |  |
| Quantitative variables | 11 | Explain how quantitative variables were handled in the analyses. If applicable, describe which groupings were chosen, and why | Pages 6-7 |  |  |
| Statistical methods | 12 | (a) Describe all statistical methods, including those used to control for confounding  (b) Describe any methods used to examine subgroups and interactions  (c) Explain how missing data were addressed  (d) *Cohort study* - If applicable, explain how loss to follow-up was addressed  *Case-control study* - If applicable, explain how matching of cases and controls was addressed  *Cross-sectional study* - If applicable, describe analytical methods taking account of sampling strategy  (e) Describe any sensitivity analyses | Pages 6-8 |  |  |
| Data access and cleaning methods |  | .. |  | RECORD 12.1: Authors should describe the extent to which the investigators had access to the database population used to create the study population.  RECORD 12.2: Authors should provide information on the data cleaning methods used in the study. | Page 4, Appendix Figure S1 |
| Linkage |  | .. |  | RECORD 12.3: State whether the study included person-level, institutional-level, or other data linkage across two or more databases. The methods of linkage and methods of linkage quality evaluation should be provided. | Page 4-5 |
| **Results** | | | | | |
| Participants | 13 | (a) Report the numbers of individuals at each stage of the study (*e.g.*, numbers potentially eligible, examined for eligibility, confirmed eligible, included in the study, completing follow-up, and analysed)  (b) Give reasons for non-participation at each stage.  (c) Consider use of a flow diagram | Appendix Figure S1 | RECORD 13.1: Describe in detail the selection of the persons included in the study (*i.e.,* study population selection) including filtering based on data quality, data availability and linkage. The selection of included persons can be described in the text and/or by means of the study flow diagram. | Appendix Figures S1 |
| Descriptive data | 14 | (a) Give characteristics of study participants (*e.g.*, demographic, clinical, social) and information on exposures and potential confounders  (b) Indicate the number of participants with missing data for each variable of interest  (c) *Cohort study* - summarise follow-up time (*e.g.*, average and total amount) | Pages 8-9, Table 1, Appendix Table S1 &S2 |  |  |
| Outcome data | 15 | *Cohort study* - Report numbers of outcome events or summary measures over time  *Case-control study* - Report numbers in each exposure category, or summary measures of exposure  *Cross-sectional study* - Report numbers of outcome events or summary measures | Appendix Tables S3-5 |  |  |
| Main results | 16 | (a) Give unadjusted estimates and, if applicable, confounder-adjusted estimates and their precision (e.g., 95% confidence interval). Make clear which confounders were adjusted for and why they were included  (b) Report category boundaries when continuous variables were categorized  (c) If relevant, consider translating estimates of relative risk into absolute risk for a meaningful time period | Pages 9-11, Appendix Table S3-5, Figures 1-3, Appendix Figures S2-4 |  |  |
| Other analyses | 17 | Report other analyses done—e.g., analyses of subgroups and interactions, and sensitivity analyses | Appendix Figures S5-S8 |  |  |
| **Discussion** | | | | | |
| Key results | 18 | Summarise key results with reference to study objectives | Page 12 |  |  |
| Limitations | 19 | Discuss limitations of the study, taking into account sources of potential bias or imprecision. Discuss both direction and magnitude of any potential bias | Pages 15-16 | RECORD 19.1: Discuss the implications of using data that were not created or collected to answer the specific research question(s). Include discussion of misclassification bias, unmeasured confounding, missing data, and changing eligibility over time, as they pertain to the study being reported. | Page 15-16 |
| Interpretation | 20 | Give a cautious overall interpretation of results considering objectives, limitations, multiplicity of analyses, results from similar studies, and other relevant evidence | Pages 12-15 |  |  |
| Generalisability | 21 | Discuss the generalisability (external validity) of the study results | Page 15 |  |  |
| **Other Information** | | | | | |
| Funding | 22 | Give the source of funding and the role of the funders for the present study and, if applicable, for the original study on which the present article is based |  |  |  |
| Accessibility of protocol, raw data, and programming code |  | .. |  | RECORD 22.1: Authors should provide information on how to access any supplemental information such as the study protocol, raw data, or programming code. | codes are in the code files in online repository |

Pages refer to the original, word document submission.

1. Barker MM, Davies MJ, Sargeant JA, Chan JCN, Gregg EW, Shabnam S, et al. Age at Type 2 Diabetes Diagnosis and Cause-Specific Mortality: Observational Study of Primary Care Patients in England. Diabetes Care. 2023;46(11):1965-72.
